# Change in composition and potential functional genes of microbial communities on carbonatite rinds with different weathering times

**DOI:** 10.3389/fmicb.2022.1024672

**Published:** 2022-11-01

**Authors:** Jin Chen, Fangbing Li, Xiangwei Zhao, Yang Wang, Limin Zhang, Lingbin Yan, Lifei Yu

**Affiliations:** ^1^Key Laboratory of Plant Resources Conservation and Germplasm Innovation in Mountainous Region (Ministry of Education), College of Life Sciences and Institute of Agro-Bioengineering, Guizhou University, Guiyang, Guizhou, China; ^2^Institute of Guizhou Mountain Resources, Guizhou Academy of Sciences, Guiyang, Guizhou, China

**Keywords:** ecological succession, biodeterioration, bioweathering, r strategy, K strategy, functional genes

## Abstract

Organisms and time are important factors for rock weathering to form soils. However, weathering time is usually difficult to quantitatively study, and the potential microorganisms involved in rock weathering are difficult to identify qualitatively. Currently, there is no clear conclusion on how ecological strategies of carbonatite weathering rind microorganisms change with weathering time, and how the microbial composition and functional genes involved in element cycling change over two century-scale weathering time. In this study, we selected abandoned carbonate tombstones as the subject and used the date when the tombstones were erected by humans as the onset of weathering. Using metagenome sequencing methods, we investigated the trends in the composition of fungal, bacterial and archaeal communities of carbonate weathering rind and related elemental cycle functional genes during a weathering time of 19 to 213 years. The results showed that: (1) with the increase in weathering time, at the phylum level, microbial taxa gradually shifted from r-strategists (faster turnover rates, higher mortality rates, higher reproduction, lower competition rate) to K-strategists (slower turnover rates, lower mortality rates, lower reproduction, higher competition rate), which correspondingly increased the abundance of functional genes related to C and N cycles. (2) The properties of the parent rock layer determines the colonization and distribution of weathering rind microorganisms (especially prokaryotic microorganisms) and the corresponding functional gene abundance. Our study provides new insights into the weathering process of carbonate rocks.

## Introduction

Rock weathering is the result of a combination of physical, chemical, and biological weathering, with organisms, particularly microorganisms, playing an important role in the early rock weathering process. On one hand, biodeterioration of rocks caused by microbial activity has led to extensive destruction of artefacts, and therefore biodeterioration of rocks is considered to be one of the most important factors threatening the safety of stone artefacts and buildings (Liu et al., 2020). On the other hand, in degraded ecosystems (e.g., karstic desertification zones), the interaction of microorganisms with rock minerals produces protons, hydroxyl ions, or metal-chelating metabolic products that alter the dynamics of rock surface reactions (Rogers and Bennett, 2004), facilitate weathering processes and form soils that are important contributors to the ecological restoration of degraded areas. As a result, the biological weathering of rocks has received widespread attention (Warscheid and Braams, 2000; Gaylarde and Gaylarde, 2004; Checcucci et al., 2022; Liu et al., 2022; Wu et al., 2022). The biological deterioration of rocks and lithic artefacts is a complex process of microbial action in which a rich diversity of microorganisms are involved in a variety of biogeochemical cycling processes (Sáiz-Jiménez, 1999; Warscheid and Braams, 2000; Gaylarde and Gaylarde, 2004; Gadd, 2017; Liu et al., 2020). Therefore, the use of microbiomics to decipher the structure and function of microorganisms involved in rock weathering is of great ecological importance.

Bioreceptivity originated from materials science, which is the ability of materials to be colonized by living organisms (Guillitte, 1995). Besides, the bioreceptivity of rocks is affected by environmental conditions (such as temperature, humidity and light, etc.) and rock properties (such as porosity, water permeability and roughness, etc.) (Miller et al., 2012). Differences in the bioreceptivity of rocks allow different kinds of microscopic taxa (such as bacteria, fungi, algae, etc.) to deposit on the rock surface, forming biofilms (Warscheid and Braams, 2000). In the beginning, the structural pores of the rock surface provided suitable niches for the dissemination of airborne microbes for their deposition and capture (Šimonovičová et al., 2004). Due to the very limited availability of nutrients on the rock surface, carbon dioxide in the air is the main carbon source for microbes on the rock surface (Liu et al., 2020). Phototrophs (such as cyanobacteria and algae, etc.) and chemolithoheterotroph (nitrifying bacteria and sulfur-oxidizing fungi, etc.) are major contributors to the assimilation of carbon dioxide into organic form for subsequent bio-colonizers (Salvadori and Municchia, 2016; Vázquez-Nion et al., 2018). Ammonia and ammonia oxides in the air are the main nitrogen sources for the microorganisms on the rock surface, which are utilized by nitrifying bacteria and archaea to produce nitrous oxide and nitric acid, which cause acid corrosion of stone artefacts (Meng et al., 2016, 2017). In addition, sulfur exists in the atmosphere in organic and inorganic forms of various valence states, and in the sulfur cycle, sulfur-oxidizing bacteria and sulfate-reducing bacteria are important factors leading to biocorrosion of rocks (Kusumi et al., 2011, 2013). However, the above studies are mainly aimed at the study of rock surface microorganisms in a certain period of time, and very few studies involve the succession of microbial communities on the surface of carbonate rocks and their correlation with the dissolution characteristics of carbonate rocks on a time scale.

From a soil science perspective, rock biodeterioration is the process of biological decomposition of rock minerals to form soil, which is manifested by surface disintegration (Gleeson et al., 2006), loss of rock hardness, and accumulation of secondary minerals. From an ecological point of view, rock biodeterioration is a process of biological primary succession on the rock surface, manifested by biofilm attachment, covering of the rock surface with green-black stains (Cutler et al., 2013b), and formation of bio-pitting (Piñar et al., 2013), which, in turn, alters the rock surface environment and supports the growth of lichens and bryophytes. Succession theory can be applied to the process of rock succession, and in Odum’s related account of plant community succession, r-strategists dominate early in succession, while K-strategists dominate later (Odum, 1969). In a stable environment, the reproduction of organisms is likely to reach the environmental capacity (that is, the saturation density K value in the logistic equation), while in an unstable environment, disturbances often occur, with high reproductive capacity (innate rate of increase) organisms are usually able to adapt (MacArthur, 1960; MacArthur and Wilson, 1967). K-strategists usually have slower growth rate and higher competitive capacity, but r-strategists usually with a faster growth rate and higher turnover rate (Bardgett et al., 2005; Yan et al., 2020). The reproduction rate of bacteria is higher than that of fungi, and the competition rate of fungi is higher than that of bacteria. Therefore, in microbial communities, fungi tend to be K-strategists, while bacteria tend to be r-strategists (Bardgett et al., 2005; Kaiser et al., 2014; Chen et al., 2016). During secondary succession in derelict land, the ratio of fungi to bacteria increases, indicating a gradual increase in the number of K-strategists during secondary succession (Zhou et al., 2017). In addition, within bacterial taxa, Acidobacteria and Actinobacteria are considered K-strategists, while Proteobacteria and Bacteroidetes are considered r-strategists (Fierer et al., 2007; Zechmeister-Boltenstern et al., 2015). Among the fungal taxa, Basidiomycota is considered a K-strategist, while Ascomycota is considered an r-strategist (Bastian et al., 2009; Zumsteg et al., 2012). In archaeal communities, Euryarchaeota are thought to occur early in the succession, while Crenarchaeota are thought to occur later (Zumsteg et al., 2012). Further, the abundance of genes associated with C (for example, *rbcL, accA* and *porA* etc.) and N (such as *nifH*, *nifK* and *napA* etc.) cycling has been reported to be greater in late- rather than in the early succession (Blaud et al., 2018; Liu et al., 2018; Yan et al., 2020). However, the strategic shift in microbial taxa and trends in functional genes during weathering of carbonate rocks are not well understood.

Southwest China is a typical karst distribution area, where carbonate rock is widely used as a common building material for tombstones. According to local customs, when a tombstone is built, the time of its erection will be engraved at the same time, which provides us with a relatively accurate weathering start time. Considering the possible damage caused by sampling, we chose to investigate abandoned tombstones where tomb relocation events have occurred. We calculated the carbonatite weathering duration (2021 minus the engraved year on the tombstone) using the etched date on the abandoned tombstone as the weathering start time. This study investigated the bioweathering patterns of carbonate rocks on a time scale using abandoned carbonate rocks at different times of monument erection, which helps to further our understanding of microbial weathering processes and mechanisms of carbonate rocks. During progressive succession, environmental conditions tend to change from unstable to stable, and from entropy increase to entropy decrease. With the increase of weathering time, the surface of carbonatite is dissolved (Zha et al., 2020), roughness increases (Emmanuel and Levenson, 2014; Levenson and Emmanuel, 2016), and many microhabitats are formed (Jones, 1965), which increases the accumulation ability of organic matter in the atmosphere (Saheb et al., 2016), the environment tends to be stable, and the carbonatite rock rind begins to form. The carbonatite surface gradually became suitable for the r-strategist from the initial suitable for the K-strategist. Therefore, we hypothesized the following: (i) as weathering time increases, the microbial community shifts from r-strategy (i.e., high turnover, low competition) to K-strategy (i.e., high competitiveness, low growth). (ii) With the increase in weathering time, the abundance of functional genes related to C, N, and S cycles gradually increases.

To test the hypotheses, we selected carbonatite tombstones concentrated in a small area, with little disturbance, and different weathering times as the research objects. We used metagenomes to interpret the structure and functional composition of microbial communities to explore the changing trend of microbial community structure and function during bioweathering of carbonate rocks during succession. Our research goal is to better describe and understand the microbial weathering patterns of carbonate rocks during succession.

## Materials and methods

### Site description and sampling strategy

The study area is located in the karst area of southwest China near the Guiyang City (26°26′46″–26°26′56″, 106°38′06″–106°39′18″), which has a subtropical humid and mild climate, with rain and heat in the same period. The average annual temperature is 15.3°C, the average altitude is about 1,100 m, the average annual rainfall is approximately 1,100–1,300 mm, and the sunshine duration are about 1,000–1,100 h.

**Figure 1:**
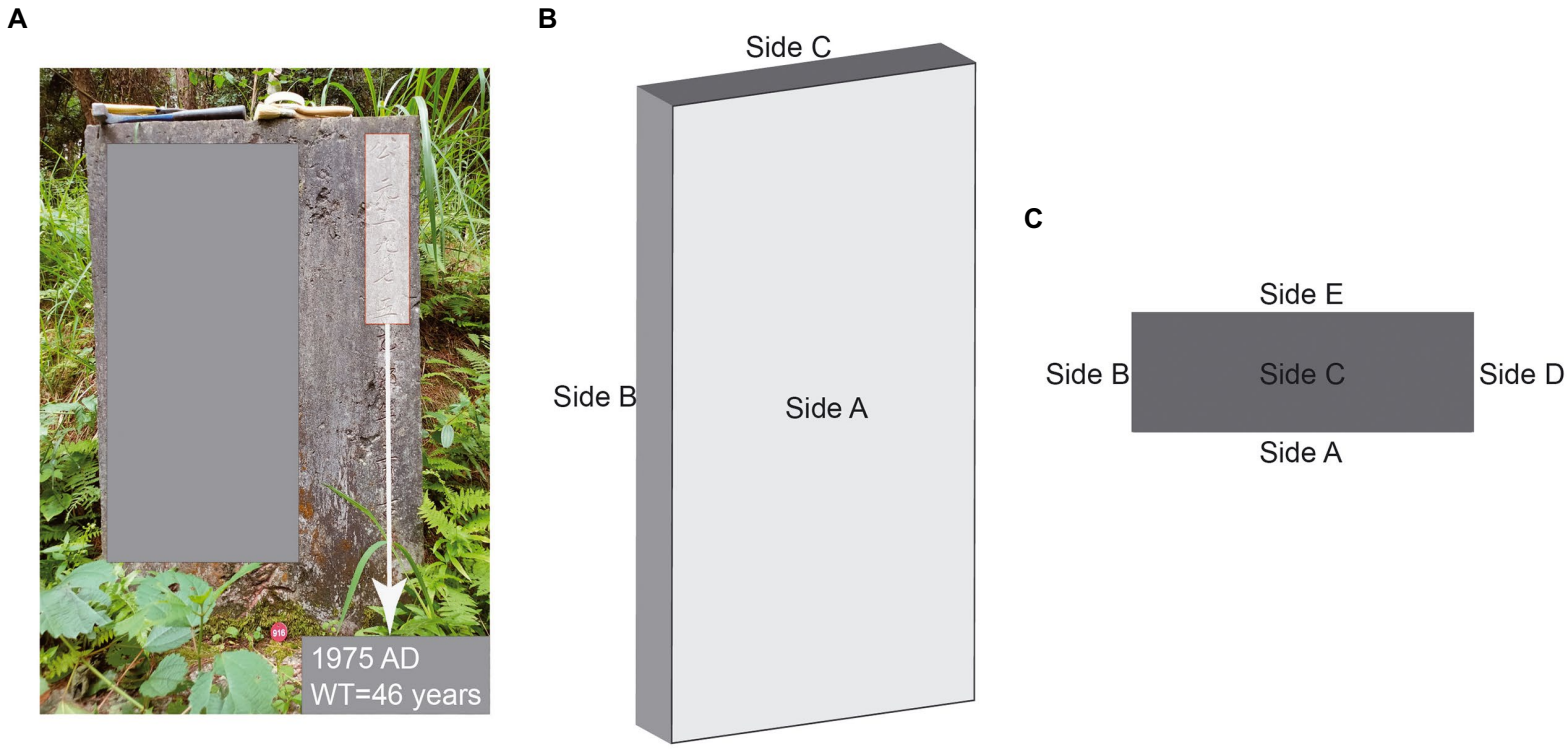
Sketch of carbonatite tombstone; **(A)** Entity diagram of carbonatite tombstone, **(B)** Stereogram of carbonatite gravestone, **(C)** Top view of carbonatite tombstone.

We used the tombstone erection time as the onset of weathering (Figure 1A), and among the 18 abandoned tombstones we investigated, the weathering time ranged from 19 to 213 years (Supplementary Table S1). During our investigation, we determined whether the target object was carbonatite based on the results of the “cold acid test” (Ford and Williams, 2013) combined with XRD data (Supplementary Figure S1). Due to local customs, the A, B, C, and D sides of tombstones (Figure 1) are generally disturbed to varying degrees during special festivals (such as the Ching Ming Festival), while the side E is left undisturbed (Figures 1B,C). To ensure rationality of sampling, therefore, we used only the E side of tombstones in our study. In addition, the tombstones were covered with moss and/or lichens, which were carefully removed to prevent contamination of the rock samples. To obtain a true weathered crust sample, we controlled the angle grinder to ensure full contact with the rock until an entire available weathered surface was removed. To prevent cross-contamination, we replaced the grinding disc for each tombstone. For sampling, we laid a sterilized (121°C, 103 kPa for 20 min) rectangular piece of medical surgical wrap flat on the E side directly below to collect sample particles. We measured the available length and height (width) of the E side of the tombstone and recorded it (Supplementary Table S1). Before the formal sampling, we simulated the sampling situation of the angle grinder on the limestone rock surface, and obtained that the angle grinder contacted the rock surface for about 3 s, and the rock surface could be ground off. Sampling was carried out from top to bottom using an angle grinder. To collect the samples, we controlled the angle grinder to touch the E side of the tombstone at a constant speed for approximately 3 s and then moved to the next sampling points until the entire available E side was collected. Finally, the samples that fell onto the wrapping cloth were collected. For specific sampling volume and sampling area, please refer to the Supplementary Table S1. In addition, during sampling, we controlled that the lowest sampling height of the tombstone was 20 ~ 30 cm from the ground, while the highest sampling distance depended on the height of the tombstone. Finally, as a control for the regolith, we took 18 un-weathered samples of each tombstone. We used a geological hammer to knock it off and cut off the weathered part of the rock to get the un-weathered part, which is then ground into powder.

### Regolith sampling and measurement

The collected weathering rind samples (n = 18) were divided into two parts, one was passed through a 0.25-mm sieve for Organic carbon (OC), total nitrogen (TN), total phosphorus (TP), and total potassium (TK) tests, and the other was stored at −80°C after removing large sand and plant residues for metagenomic analysis. OC was determined by the H_2_SO_4_-K_2_Cr_2_O_7_ method (Bao, 2000), TN by Kjeldahl (Page, 1982), TP by the H_2_SO_4_-HClO_4_ abatement colorimetric method, and TK by flame photometry (Bao, 2000). We used the weathering strength (*W_s_*) (Huang et al., 1996) to measure the degree of weathering relative to the parent rock, and used the mobiles index (*I_mob_*) to characterize the element migration of the overweathered layer relative to the parent rock during the carbonatite weathering process (Irfan, 1996). In addition, X-ray fluorescence (XRF) was used to measure the content of major element chemistry of carbonatite, and X-ray diffraction (XRD) was used to determine the rock phase. Prior to this, in order to prevent organic matter content in the sample from affecting the test results, we placed samples in a porcelain crucible and heated them to 950°C to eliminate this effect. Finally, according to the definition of bioreceptivity (Guillitte, 1995), we took the number of all individuals in each sample as the bioreceptivity of the sample.

### DNA extraction, library construction, and metagenomic sequencing

Total genomic DNA was extracted from carbonatite regolith samples using the E.Z.N.A.® Soil DNA Kit (Omega Bio-tek, Norcross, GA, U.S.) according to manufacturer’s instructions. Concentration and purity of extracted DNA was determined with TBS-380 and NanoDrop 2000, respectively. DNA extract quality was assessed on a 1% (w/v) agarose gel.

DNA samples were fragmented to an average size of about 400 bp using Covaris M220 (Gene Company Limited, China) for paired-end library construction. A paired-end library was constructed using NEXTflex™ Rapid DNA-Seq (Bioo Scientific, Austin, TX, USA). Adapters containing the full complement of sequencing primer hybridization sites were ligated to the blunt-end of fragments. Paired-end sequencing was performed on an Illumina HiSeq Xten instrument (Illumina Inc., San Diego, CA, USA) at Majorbio Bio-Pharm Technology Co., Ltd. (Shanghai, China) using HiSeq X Reagent Kits according to the manufacturer’s instructions.^1^

### Sequence quality control and genome assembly

The raw reads from metagenome sequencing were used to generate clean reads by removing adaptor sequences, trimming, and removing low-quality reads (reads with N bases, a minimum length threshold of 50 bp and a minimum quality threshold of 20) using fastp (Chen et al., 2018; Ren et al., 2022, version 0.20.0)^2^ on the free online Majorbio Cloud Platform (cloud. majorbio.com). These high-quality reads were then assembled into contigs using MEGAHIT (Li et al., 2015) (parameters: kmer_min = 47，kmer_max = 97，step = 10, version 1.1.2)^3^ which makes use of succinct de Bruijn graphs. Contigs equal to or over 300 bp in length were selected as the final assembled result.

### Gene prediction, taxonomy, and functional annotation

Open reading frames (ORFs) in contigs were identified using MetaGene (Noguchi et al., 2006).^4^ Predicted ORFs equal to or over 100 bp in length were retrieved and translated into amino acid sequences using the NCBI translation table.^5^

A non-redundant gene catalog was constructed using CD-HIT (Fu et al., 2012, version 4.6.1)^6^ with 90% sequence identity and 90% coverage. After quality control, reads were mapped to the non-redundant gene catalog with 95% identity using SOAPaligner (Li et al., 2008, version 2.21),^7^ and gene abundance in each sample was evaluated.

Representative sequences of non-redundant gene catalogs were annotated based on the NCBI NR database using blastp as implemented in DIAMOND v0.9.19 with an e-value cutoff of 1e^−5^ using DIAMOND (Buchfink et al., 2015, version 0.8.35)^8^ for taxonomic annotations. Cluster of orthologous groups of proteins (COG) annotation for the representative sequences were performed using DIAMOND (Buchfink et al., 2015, version 0.8.35)^9^ against the eggNOG database (version 4.5.1) with an e-value cutoff of 1e^−5^. KEGG annotations were also generated using DIAMOND (Buchfink et al., 2015, version 0.8.35)^10^ against the Kyoto Encyclopedia of Genes and Genomes database (version 94.2)^11^ with an e-value cutoff of 1e^−5^.

### Calculations and statistical analyses

The abundance counts for taxa were converted by multiplying the relative abundance by the minimum total sequence count for all samples, and this method was used to correct for the different sequencing depths of the samples (McMurdie and Holmes, 2014; Weiss et al., 2017). The relative abundance of a bacterial phylum is the ratio of the abundance of each bacterium at the phylum level compared to the total bacterial abundance. In addition, the relative abundance of fungal taxa, archaeal taxa, and functional genes is determined in the same way as bacterial taxa. Functional gene abundance is often used to predict biogeochemical cycle rates (Jha et al., 2017). For example, *nifH* for N2 fixation (Rösch and Bothe, 2005) and *amoA* for nitrification (Wei et al., 2019). We find the relevant KO numbers based on the functional genes associated with a particular biogeochemical cycle process as the corresponding functional genes. For example, in the N cycle, the KO number of *nifH* is K02588, while the *nifK* is K02591, and both K02588 and K02591 are components of N-cycle corresponding functional genes (see Supplementary Table S2 for more details).

The relative abundance of C, N, and S cycle-related genes was calculated as the ratio of C, N, and S cycle-related gene abundance compared to the KEGG Orthology total gene abundance. The relative abundance of C Fixation genes, C Degradation genes, and Methane Metabolism genes were calculated based on the ratios of C Fixation genes, C Degradation genes, and Methane Metabolism genes to the abundance of C cycle genes, respectively. The relative abundance of Calvin cycle genes, Reductive citrate cycle (rTCA) genes, Reductive acetyl-CoA or Wood-Ljungdahl pathway (rAcCoA) genes, 3-Hydroxypropionate bi-cycle (3HP) genes, Hydroxypropionate-hydroxybutylate cycle (3HP/4HB) genes, and Dicarboxylate-hydroxybutyrate cycle (DC/4HB) genes were calculated based on the ratios of each respective gene compared to the abundance of C Fixation genes. Similarly, the relative abundance of Starch Degradation genes, Cellulose Degradation genes, Chintin Degradation genes, Hemicellulose Degradation genes, Lignin Degradation genes, and Aromatics Degradation genes were calculated based on the ratios of each respective gene compared to the abundance of C Degradation genes. Moreover, the relative abundance of Assimilatory N Reduction (ANRA) genes, Dissimilatory N Reduction (DNRA) genes, Nitrification genes, Denitrification genes, N Transport genes, N Fixation genes, and Organic N Metabolism (ONM) genes were also calculated based on the ratios of each respective gene compared to the abundance of N cycle genes. Further, the relative abundance of Assimilatory sulfate reduction (ASR) genes, Dissimilatory sulfate reduction (DSR) genes and Thiosulfate oxidation by SOX complex (SOX) genes were calculated based on the ratios of each respective gene compared to the abundance of S cycle genes.

The meteorological data came from the China Meteorological Administration.^12^ To analyze the characteristics of taxa and functional genes with weathering time, the significance of linear models was tested with the built-in function ‘lm’ in *stat* package of R (version 4.2.1). *p* values were obtained by permutation test when the data did not conform to normal distribution or/and homogeneity of variance. In the Mantel test, taxa data that exist in all samples (n = 18) were selected for analysis. The Bray–Curtis distance algorithm was used to conduct Mantel tests on taxa and functional gene data, and the Euclidean distance algorithm was used to conduct Mantel tests on environmental data. Mantel validation and drawing were performed using the R package *linkET*. When doing regression analysis, composition data were transformed by log_10_(*x* + *x*_0_), where *x* is the relative abundance, *x*_0_ is a constant, and *x*_0_ = 0.9·min(*x*). The structural equation model (SEM) was evaluated with the built-in ‘sem’ function in the R package *lavaan* (Rosseel, 2012).

## Results

### Changes in element content of carbonatite regolith at different weathering times

**Figure 2:**
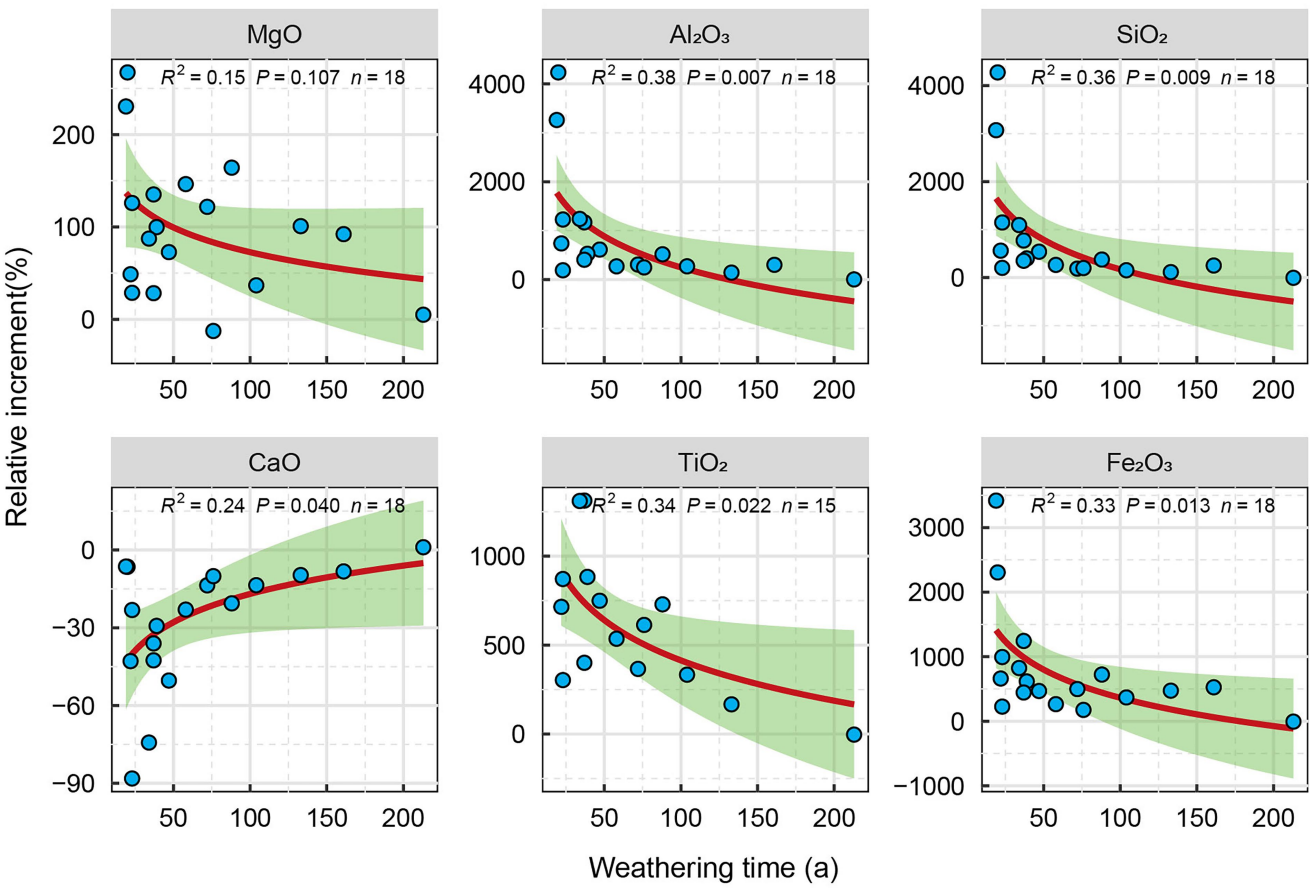
Relative variation of elements in carbonatite weathering rind with different weathering years.

Because the properties of carbonatite parent rocks at different weathering times are different, the relative changes in the element content of the regolith relative to the parent rock layer can better reflect the relative changes of the main elements of carbonatite at different weathering times. The XRF results show that, relative to the parent rock, with the increase of the carbonatite weathering time, the Mg, Al, Si, Fe, and Ti in the carbonatite regolith were gradually enriched, and the increment relative to the parent rock layer was significantly reduced (Figure 2). The content of Ca gradually decreased with the increase of weathering time, and the increment of Ca relative to the parent rock decreased significantly with the increase of weathering time (Figure 2).

**Figure 3:**
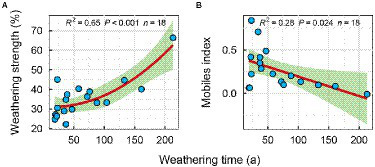
Regression fitting between weathering index and weathering time; **(A)** Weathering strength, **(B)** Mobiles index.

In addition, the mobiles index decreased significantly with increasing weathering time (Figure 3B), while the weathering strength was reversed (Figure 3A). It shows that with the increase of weathering time, the weathering degree of carbonatite gradually increases, while the element migration rate decreases gradually.

### Changes in gene abundance in microorganisms

A total of 6,282,480 non-redundant genes were screened out in the metagenomes of all samples, including 5,207,594 in bacteria, 108,547 in fungi, and 30,221 in archaea. In addition, NR annotation results showed that the ratio of fungal abundance to bacterial abundance decreased significantly with an increase in the weathering time of carbonate rocks, while the ratio of archaeal abundance to bacterial abundance did not change significantly (Supplementary Figure S2). The Shannon–Wiener index showed that the diversity of bacteria decreased significantly with an increase in weathering time, while the diversity of fungi and archaea did not change significantly with an increase in weathering time (Supplementary Figure S3). The bioreceptivity and total diversity first increased and then decreased with the increase in weathering time (Supplementary Figure S2).

### Changes in fungal, bacterial, and archaeal diversity and composition at different weathering times

**Figure 4:**
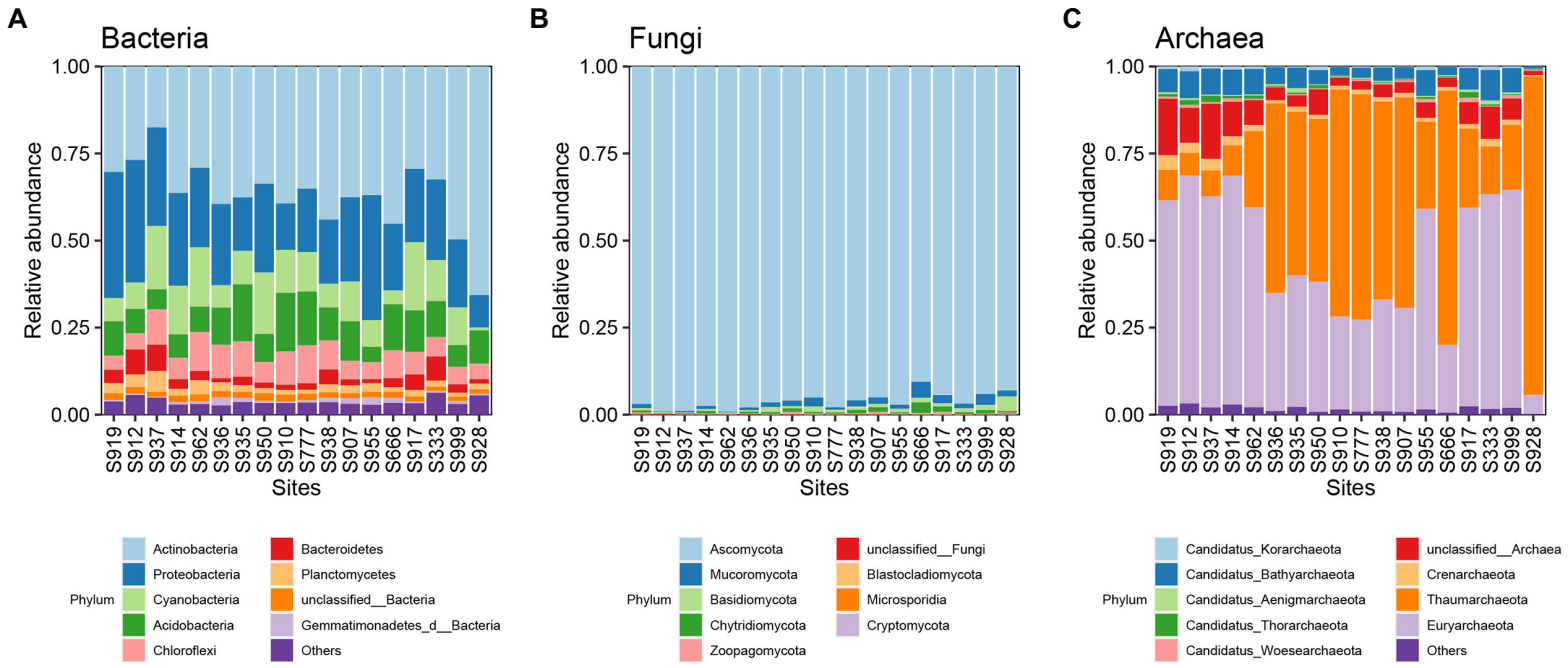
The relative abundance of different taxa at the phylum taxonomic level (the top 10 relative abundances are shown); **(A)** Bacteria, **(B)** Fungi and **(C)** Archaea.

Based on similarities to entries in the NCBI-NR database, among the 18 samples, per sample read counts ranged from 18,326 to 22,469 reads, 97.88% of sequences were classified as Bacteria, 0.41% were classified as Archaea, 1.51% were classified as Eukaryota (among them, 1.21% were classified as Fungi), and 0.03% were classified as Viruses. The phyla Actinobacteria (37.03%) and Proteobacteria (23.07%) dominated carbonate rock rind bacterial communities (Figure 4). The average relative abundance of Actinobacteria significantly increased with the increase in weathering time (Table 1). The average relative abundance of Proteobacteria decreased significantly with the increase in weathering time, while the other bacterial phyla with average relative abundance >1% had no significant trend during succession (Table 1). The phyla Ascomycota (96.25%) dominated carbonate rock rind fungal communities (Figure 4). The relative abundance of Ascomycota decreased significantly, while Mucoromycota and Basidiomycota increased significantly during succession (Table 1). The phyla Euryarchaeota (44.28%) and Thaumarchaeota (38.49%) dominated carbonate rock rind archaeal communities (Figure 4). At a threshold of mean relative abundance >1%, Crenarchaeota and Euryarchaeota decreased significantly, while Thaumarchaeota showed no significant change during succession (Table 1).

**Table 1:** The variations of phyla of bacteria, fungi, and archaea over different weathering times (average relative abundance >1%).

Phylum	Slope	*SE*	*p*-param	*R* ^2^	Adj *R*^2^	HmV and Normality	*p*-perm	*p*-sign
Acidobacteria	−2.12E-05	7.48E-04	0.978	<0.01	Null	Null	Null	0.978
Actinobacteria	1.41E-03	4.22E-04	0.004	0.41	0.38	No, Non-N	0.005	0.005^**^
Bacteroidetes	−8.07E-04	1.21E-03	0.516	0.03	Null	Null	Null	0.516
Chloroflexi	−1.18E-03	6.14E-04	0.072	0.19	Null	Null	Null	0.072
Cyanobacteria	−3.20E-03	1.20E-03	0.017	0.31	0.26	No, HtV	0.029	0.029^*^
Planctomycetes	−1.71E-03	7.60E-04	0.039	0.24	0.19	Yes	Null	0.039^*^
Proteobacteria	−1.50E-03	5.81E-04	0.020	0.29	0.25	Yes	Null	0.020^*^
Ascomycota	−1.19E-04	3.60E-05	0.005	0.40	0.37	No, Non-N	0.012	0.012^*^
Basidiomycota	3.89E-03	7.94E-04	0.000	0.60	0.57	Yes	Null	<0.001^***^
Mucoromycota	3.59E-03	1.45E-03	0.025	0.28	0.23	Yes	Null	0.025^*^
Candidatus Bathyarchaeota	−2.39E-03	1.10E-03	0.045	0.23	0.18	No, HtV	0.057	0.057
Crenarchaeota	−2.33E-03	8.72E-04	0.017	0.31	0.27	Yes	0.017	0.017^*^
Euryarchaeota	−2.44E-03	1.07E-03	0.036	0.25	0.20	No, HtV	0.037	0.037^*^
Thaumarchaeota	2.12E-03	1.66E-03	0.221	0.09	Null	Null	Null	0.221

The relative abundance of bacterial, fungal, and archaeal phyla were averaged for each sample (*n* = 18). The variance explained (*R*^2^), regression slope, and parametric *p* value (*p*-param) of the linear regression, normality test, homogeneity test for variance, and permutation test *p* value (*p*-perm) with different weathering times are shown in the table (^*^indicates *p* < 0.05, ^**^ indicates *p* < 0.01, and ^***^indicates *p* < 0.001). HmV, homogeneity of variance; HtV, heterogeneity of variance; *p*-sign, significance value of *p*.

### Potential functional pathways in microorganisms at different weathering times

The corresponding ORFs were annotated according to the KEGG and CAZy databases to quantify the changes of potential functional genes of carbonate rock rind at different weathering times. In total, 450 pathway-associated genes and 552 CAZy genes were screened from all metagenomes. The dominant categories were biosynthesis of secondary metabolites (8.41%), microbial metabolism in diverse environments (5.67%), biosynthesis of amino acids (3.05%), carbon metabolism (2.89%), ABC transporters (2.73%), and quorum sensing (1.8%) of the total KEGG pathway Level 3 annotated genes (Supplementary Table S3). In all pathways with average relative abundance >1%, biosynthesis of secondary metabolites, microbial metabolism in diverse environments, biosynthesis of amino acids, carbon metabolism, glyoxylate and dicarboxylate metabolism, and pyruvate metabolism increased significantly with an increase in weathering time (Supplementary Table S3). However, ABC transporters, quorum sensing, and two-component systems significantly decreased with an increase in weathering time, while the other pathways showed no significant changes with weathering time (Supplementary Table S3). Glycoside hydrolases (30.92%) and glycosyl transferases (38.39%) were dominant in the class hierarchy of CAZY-related enzyme genes. However, the mean relative abundance of all class levels did not change significantly with the increase in weathering time (Supplementary Table S4).

### Changes in functional genes related to C, N, and S cycle at different weathering times

We identified genes associated with C, N, and S cycles based on the KEGG Orthology database. We identified 2,640,106 genes related to the C cycle, 1,062,634 genes related to the N cycle, and 550,188 genes related to the S cycle in all metagenomes. During succession, we found that the relative abundance of genes related C (from 2.21 to 2.75%) and N (from 0.83 to 1.22%) cycling significantly increased with time (Table 2). However, the relative abundance of genes related to S cycling did not change significantly with time (Table 2). Furthermore, in S cycling, we found that the relative abundance of genes related to ASR (from 90.07 to 98.01%) significantly increased with time, but SOX (from 8.91 to 1.51%) decreased (Table 2). In N cycling, the relative abundance of genes related to denitrification (from 5.58 to 0.43%) and nitrification (3.34 to 0.06%) significantly decreased with time (Table 2), while, the other N cycle processes showed no significant changes with time. In C cycling, the relative abundance of genes related to C degradation (from 17.51 to 10.31%) significantly decreased with time, while genes related to C Fixation and methane metabolism showed no significant trend with time (Table 2). In C Fixation, the relative abundance of genes related to 3HP/4HB (from 25.14 to 21.62%, with the main taxa being Sphingomonadaceae and Rubrobacteraceae; Supplementary Figure S12) and 3HP (from 26.89 to 22.71%, with the main taxa being Rubrobacteraceae and Sphingomonadaceae; Supplementary Figure S11) significantly decreased with time, while genes related to DC/4HB (from 33.93 to 42.54%), rTCA (from 43.55 to 51.97%), and rAcCoA (from 9.34 to 12.18%) significantly increased with time (Table 2). In C Degradation, the relative abundance of genes related to starch degradation (from 30.04 to 46.45%) and aromatics degradation (from 0.34 to 1.65%) significantly increased with time, while genes related to cellulose degradation (from 30.05 to 16.68%) significantly decreased with time (Table 2).

**Table 2:** Variations of the relative abundance of genes related to microbial biogeochemical cycling over different weathering times (mean relative abundance >1%).

Gene function category	Relative abundance % (mean abundance)	HmV and normality	Slope	SE	*R* ^2^	p-param	Adj *R*^2^	*p*-perm	*p*-sign
S Cycle	0.49% (30566)	Null	−2.58E-04	1.47E-04	0.16	0.099	Null	Null	Null
N Cycle	0.95% (59035)	No, HtV	8.73E-04	3.31E-04	0.30	0.018^*^	0.26	0.035	0.035^*^
C Cycle	2.34% (146672)	No, HtV	1.38E-03	3.85E-04	0.44	0.003^**^	0.41	0.012	0.012^*^
C Fixation	76.09% (111643)	Null	1.11E+00	5.51E-01	0.20	0.060	Null	Null	Null
C Degradation	12.70% (18588)	No, HtV	−1.51E+00	4.83E-01	0.38	0.007^**^	0.34	0.007	0.007^**^
Methane Metabolism	11.21% (16440)	Null	3.97E-01	3.43E-01	0.08	0.265	Null	Null	Null
ANRA	6.65% (3911)	Null	−1.44E-02	2.20E-02	0.13	0.523	Null	Null	Null
DNRA	4.36% (2592)	Null	1.29E-02	1.20E-02	0.12	0.302	Null	Null	Null
N Fixation	0.21% (122)	Null	1.96E-04	4.79E-03	0.06	0.968	Null	Null	Null
N Transport	5.10% (2990)	Null	1.75E-02	2.93E-02	0.17	0.560	Null	Null	Null
Denitrification	1.39% (804)	Yes	−3.27E-02	1.36E-02	0.55	0.002^*^	0.49	0.002	0.002^**^
Nitrification	0.65% (368)	Yes	−2.14E-02	8.12E-03	0.60	<0.001^***^	0.55	<0.001	<0.001^***^
ONM	82.14% (48528)	Null	2.15E-02	3.89E-02	0.03	0.588	Null	Null	Null
ASR	93.87% (28648)	Yes	2.16E-02	8.26E-03	0.30	0.019^*^	0.26	0.019	0.019^*^
DSR	6.67% (2019)	Null	7.26E-04	4.52E-03	0.00	0.874	Null	Null	Null
SOX	5.47% (1713)	Yes	−2.16E-02	7.60E-03	0.34	0.012^*^	0.29	0.012	0.012^*^
Hemicellulose	27.50% (5140)	Null	2.19E-01	7.66E-01	0.01	0.779	Null	Null	Null
Starch	41.47% (7620)	Yes	2.99E+00	1.24E+00	0.27	0.028^*^	0.22	0.028	0.028^*^
Aromatics	1.00% (181)	Yes	2.74E-01	1.08E-01	0.29	0.021^*^	0.24	0.021	0.021^*^
Chintin	8.04% (1532)	Null	−1.07E+00	7.26E-01	0.12	0.159	Null	Null	Null
Lignin	0.03% (6)	Null	−2.60E-02	1.35E-02	0.19	0.072	Null	Null	Null
Cellulose	21.97% (4109)	Yes	−2.38E+00	1.01E+00	0.26	0.031^*^	0.21	0.031	0.031^*^
3HP/4HB	23.86% (26699)	Yes	−1.12E-02	3.96E-03	0.34	0.012^*^	0.29	0.012	0.012^*^
3HP	25.78% (28819)	Yes	−1.34E-02	3.73E-03	0.45	0.002^**^	0.41	0.002	0.002^**^
DC/4HB	36.16% (40311)	No, HtV	2.05E-02	7.41E-03	0.32	0.014^*^	0.28	0.029	0.029^*^
rTCA	45.55% (50766)	No, HtV	1.88E-02	7.46E-03	0.28	0.023^*^	0.24	0.046	0.046^*^
rAcCoA	10.89% (12227)	Yes	3.27E-02	1.06E-02	0.39	0.008^**^	0.31	0.008	0.008^**^
Calvin cycle	27.87% (31093)	Null	−3.55E-01	2.60E-01	0.10	0.191	Null	Null	Null

The relative abundances of gene function category were averaged for each site (*n* = 18). The variance explained (*R*^2^), regression slope, and parametric *p*-value (*p*-param) of the linear regression, normality test, homogeneity test for variance, and permutation test *p* value (*p*-perm) with different weathering times were shown in the table (^*^ indicates *p* < 0.05, ^**^ indicates *p* < 0.01, and ^***^indicates p < 0.001). HmV, homogeneity of variance; HtV, heterogeneity of variance; p-sign, significance value of *p*.

### Drivers of microbial biodiversity and biogeochemical cycling genes at different weathering times

**Figure 5:**
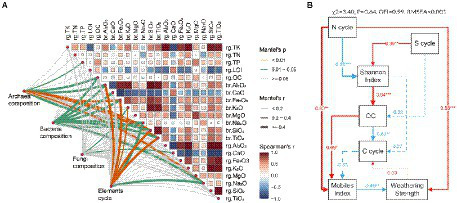
Biotic and abiotic drivers of microbial community composition and rock weathering. **(A)** Pairwise comparisons of environmental factors are shown, with a color gradient denoting spearman’s correlation coefficints. Taxonomic and functional (based on biochemical KEGG Orthologs) genes composition was related to each environmental factor of Mantel tests. Edge width corresponds to the Mantel’s r statistic for the corresponding distance correlations, and edge color denotes the statistical based on 9,999 permutations. **(B)** Structural equation modeling. The rg prefix indicates regolith and the br prefix indicates parent rock. Red and blue arrows represent positive and negative path coefficients, respectively, and dashed arrows represent non-significant paths (*p* > 0.05). The path width is proportional to the absolute value of the path coefficients, and numbers are normalized path coefficients.

To identify the drivers of carbonate rock rind microbes and related gene functions at different weathering times, we correlated differences in taxa composition (present in all 18 samples) and gene function composition (relative abundance of genes mainly related to C, N, and S cycles) with differences in environmental factors (physicochemical factors and oxide molecular ratio of regolith, and parent rock oxide molecular ratio; Figure 5A). We found an interesting phenomenon: the composition of gene abundances related to archaeal community, bacterial community, and element cycling is mostly related to the parent rock oxide ratio compared to the regolith oxide molecular ratio and physicochemical factors (Figure 5A). However, fungal community composition was insensitive to these environmental factors. In addition, the organic carbon content of the regolith was significantly correlated with the composition of archaeal and bacterial communities in the carbonatite regolith at different weathering times. Mantel analysis showed that the properties of the parent rock and the organic carbon content of the regolith may be important driving factors affecting the archaea and bacteria of the carbonate rock rind at different weathering times.

We used SEM to investigate the direct and indirect effects of functional genes related to cycling and microbial diversity in carbonate weathering regolith at different weathering times on the degree of carbonate weathering. We found that N cycle-related functional genes had significant direct positive effects on mobiles index and weathering strength. Meanwhile, the OC content of the weathering regolith has a significant direct positive effect on the mobiles index, but the mobiles index has a significant direct negative effect on the weathering strength (Figure 5B). In addition, N cycle-related functional genes had a significant direct negative effect on the Shannon diversity index, while the S cycle had a significant direct positive effect on the Shannon diversity index. Shannon diversity index had a significant direct positive effect on OC, while OC had a significant direct negative effect on C cycling, which had a direct negative effect on migration index (Figure 5B). SEM indicated that the differences in the abundance of N and S cycle-related functional genes may be the driving mechanism for the microbial diversity of carbonate rock rind at different weathering times. Additionally, the differences in the abundance of functional genes related to the N cycle may also be the driving mechanism for the natural weathering of carbonate rocks.

## Discussion

### Variation characteristics of carbonate weathering rind taxa at different weathering times

Despite rapid temperature fluctuations and poor water availability on the rock surface, many species of microorganisms have colonized (Gaylarde et al., 2017a,b; Louati et al., 2020; Ding et al., 2021). In this study, the overall abundance of microbial taxa (about 21,270 on average) was lower than that in forest soils (about 4.6 million on average) and farmland soils (about 2.23 million on average; Yan et al., 2020). In addition, the Shannon index of microbial taxa decreased significantly with the increase of weathering time (Supplementary Figure S3). Previous studies have shown that the large temperature difference on the rock surface, poor energy source and nutrient availability, and strong ultraviolet radiation limit the colonization of microorganisms (Gorbushina, 2007; Miller et al., 2012), which may also lead to a lower abundance of microbial taxa in the carbonatite weathering rinds than in the soil. In addition, the total number of reads obtained in this study was slightly lower than those obtained in previous studies (Louati et al., 2020), which may be related to our sampling location. Our sampling position was perpendicular to the ground, which may not be conducive to microbial colonisation.

**Figure 6:**
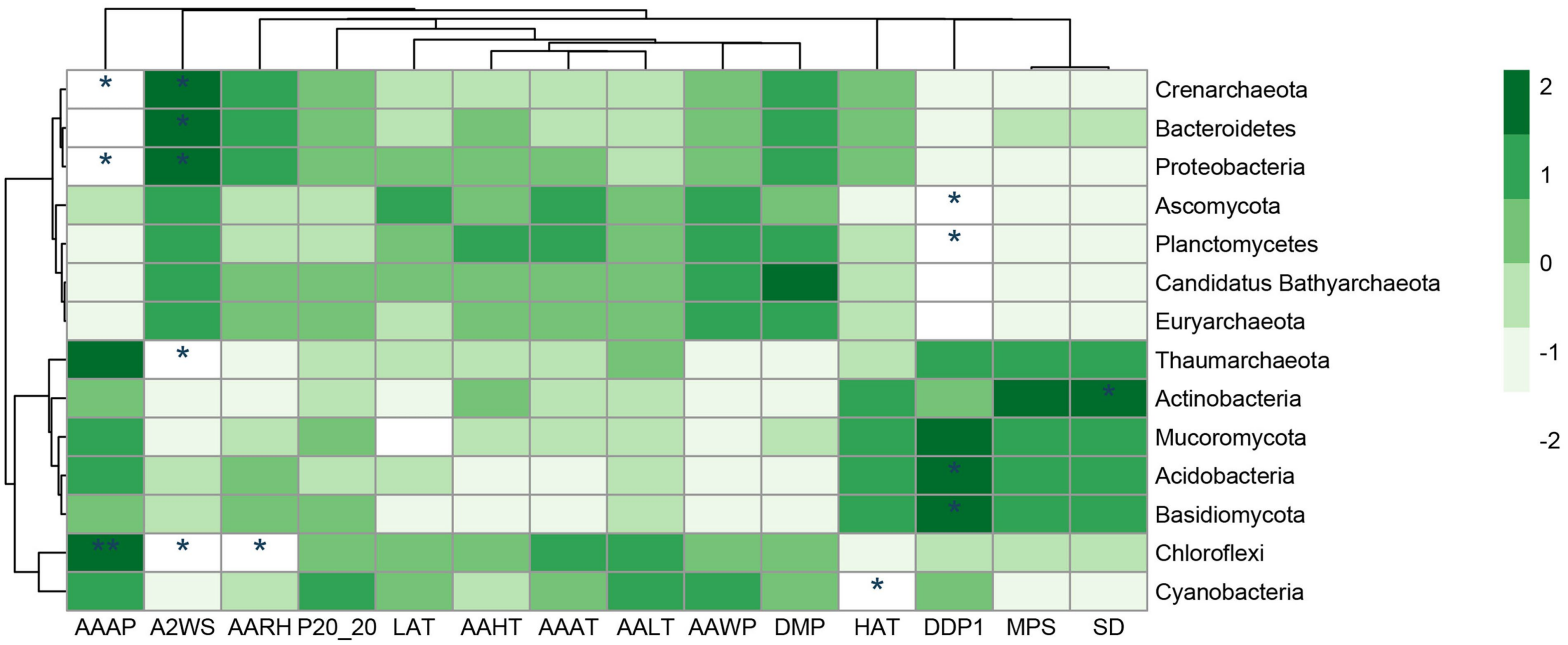
Correlation analysis between different taxa and meteorological data at phylum taxa level. AAAP, annual average atmospheric pressure (hPa); A2WS, average 2 min wind speed (m/s); AARH, annual average relative humidity (%); P20-20, recipitation of 20 p.m. − 20 p.m. (mm); LAT, the lowest air temperature (°C); AAHT, annual average highest temperature (°C); AAAT, annual average air temperature (°C); AALT, annual average lowest temperature (°C); AAWP, annual average water pressure(hPa); DMP, daily maximum precipitation (mm); HAT, the highest temperature (°C); DDP1, Days with daily precipitation > = 0.1 mm (d); MPS, monthly percentage of sunshine (%); SD, sunshine duration (h). *inidicates *p* < 0.05, **inidicates *p* < 0.01.

**Figure 7:**
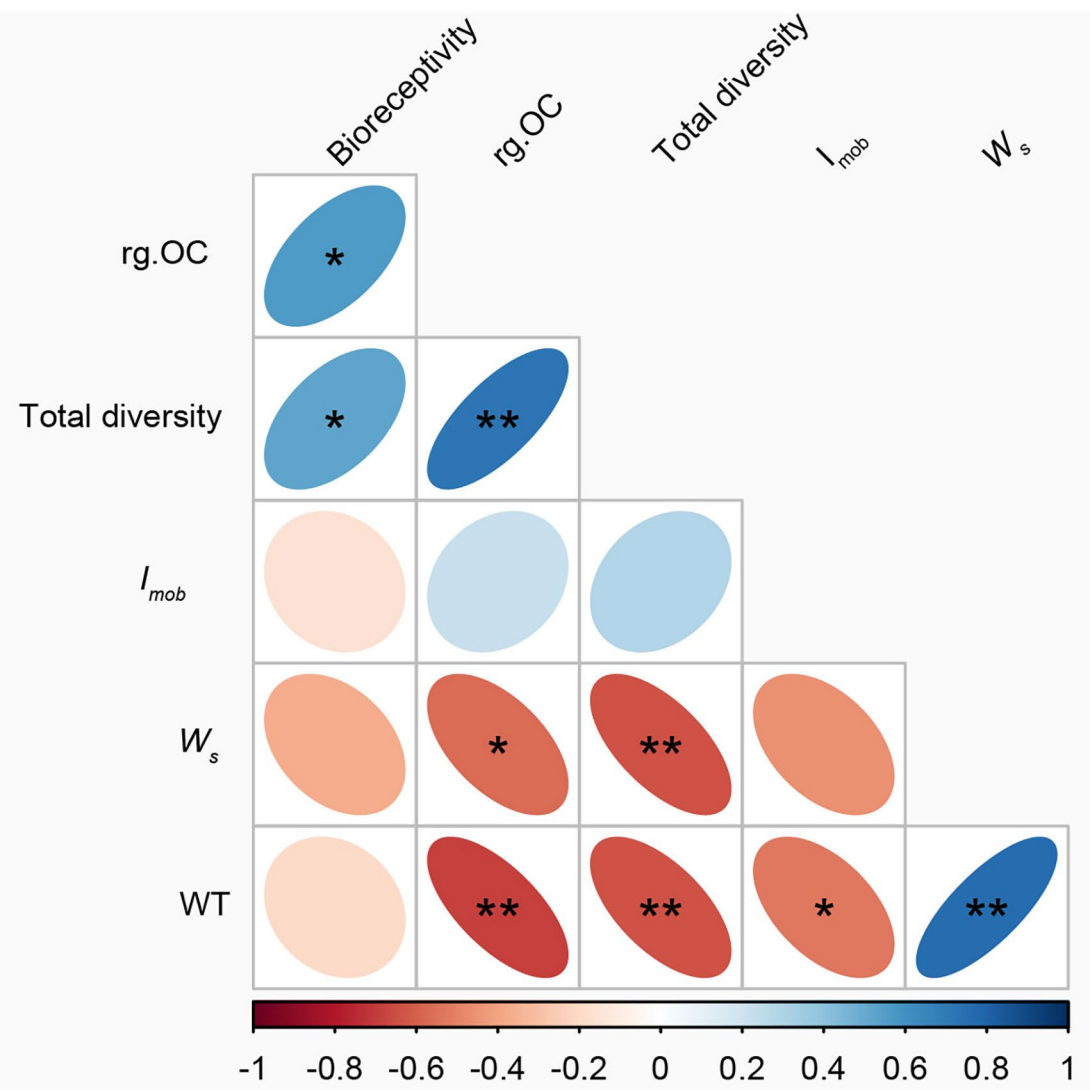
Correlation of weathering time with weathering degree, diversity index, and organic carbon. *p < 0.05, **p < 0.01.

The number of individuals first increased and then decreased with weathering time, which is different from previous studies (Nemergut et al., 2007; Zumsteg et al., 2012). This change implies that factors affecting microbial taxa are not only related to weathering time but may also be related to the rock’s parent rock composition, climatic conditions (Figure 6), nutrient status, and other factors (Lazzaro et al., 2009). In this study, bioreceptivity, total diversity, and organic carbon content were significantly positively correlated, and total diversity was significantly negatively correlated with weathering strength (Figure 7). This suggests that despite lower weathering, higher weathering rates recruit a greater variety of microorganisms, leading to higher organic carbon production rates (Figure 5B).

Regarding fungal communities, studies have shown that eukaryotic algal and fungal communities on rock surfaces tend to exhibit lower biodiversity compared to natural environments, but fungal communities on rock surfaces are more abundant and heterogeneous (Cutler et al., 2013a). Overall, the fungal community abundance decreased with weathering time. However, in the fungal community, the relative abundance of the r-strategist Ascomycota decreased significantly with weathering time, while the relative abundance of the K-strategist Basidiomycota increased significantly with weathering time; this finding is more consistent with our first hypothesis. In addition, the relative abundance of Mucoromycota also increased significantly with weathering time (Table 1), suggesting that Mucoromycota may be a K-strategist. Previous studies (Bastian et al., 2009) have shown that Ascomycota is usually suitable for living in barren environments, and is especially common as a pioneer species in the early succession; while in late succession, it is dominated by the environmental nutrient-dependent Basidiomycota. This may be because the environment is relatively barren in the early stage of carbonatite weathering, and it is more suitable for microbial colonization through nutrient enrichment in the later stage.

For bacterial communities, studies have shown that the selection of bacterial communities by petrochemical elements results in bacterial community structure driven by the chemical composition of minerals (Gleeson et al., 2006). In this study, the bacterial diversity index decreased significantly with increasing weathering time (Supplementary Figure S3). At the phylum level, the K-strategist Actinobacteria significantly increased with increasing carbonatite weathering time, while the r-strategic Proteobacteria decreased significantly with increasing weathering time (Table 1). Studies have shown that Proteobacteria are composed of a large number of phototrophs, photoheterotrophs, and chemolithotrophs, and are at an advantage in initial ecosystems with limited nutrient resources (Hodkinson et al., 2002). Actinobacteria play an important role in the decomposition of lignin and other refractory polymers (Heuer et al., 1997). In this study, Cyanobacteria, Planctomycetes, and Chloroflexi decreased significantly with weathering time, suggesting that they may be r-strategists. Cyanobacteria are photoautotrophs that form lichen symbionts with fungi and were the first to colonize bare rocks (Fermani et al., 2007). The increased C and N inputs of Cyanobacteria colonization provide an important prerequisite for the progressive succession of subsequent heterotrophs (Freeman et al., 2009).

For archaeal communities, most previous studies have reported that archaea are usually found in extreme environments (Bintrim et al., 1997; Chaban et al., 2006). Unlike bacteria and fungi, archaeal community diversity indices showed no significant change with weathering time (Supplementary Figure S3). However, Candidatus Bathyarchaeota, Crenarchaeota, and Euryarchaeota phyla decreased significantly with increasing weathering time (Table 1), which differs from previous studies. Crenarchaeota is usually a K-strategist and is enriched in late succession, while Euryarchaeota is considered to be an r-strategist and is usually present in barren environments (Zumsteg et al., 2012). Crenarchaeota has been reported to be affected by organic carbon (Zinger et al., 2011). In this study, the organic carbon content was higher in the early stage of weathering than in the later stage, which may be one of the reasons why the abundance of Crenarchaeota in the early stage of weathering was higher than that in the later stage.

### Variation characteristics of genes abundance related to C, N, and S cycling in carbonate rind at different weathering times

In this study, we found that the gene abundances of microbial taxa associated with carbonatite regolith C-cycle (the main taxa are Actinobacteria and Proteobacteria; Supplementary Figure S4A) and N-cycle (the main taxa are Actinobacteria, Proteobacteria and Cyanobacterial; Supplementary Figure S5A) increased with weathering time (Table 2), which supports our second hypothesis. The above conclusions are similar to those of the secondary succession process of plant communities (Blaud et al., 2018; Yan et al., 2020).

The abundance of C degradation-related genes decreased significantly with the increase in weathering time, which showed that the abundance of starch-related degradation genes (the main dominant taxa are *Acidobacteria bacterium, Chloroflex bacterium,* and *Kallotenue* sp., see Supplementary Figure S7B) increased significantly, while the abundance of cellulose degradation-related genes (the main dominant taxa is *Acidobacteria bacterium* and *Cellulomonas sp*., see Supplementary Figure S8B) gradually decreased significantly. This implies that during the carbonatite weathering process, the microbial niche of the carbonatite weathering crust differentiated, and the carbon source for microbial carbon degradation changed from a difficult-to-decompose carbon source (cellulose) to an easily-decomposable carbon source (starch).

In C fixation, both rTCA and rAcCoA increased significantly with weathering time, and the rTCA cycle pathway to fix CO_2_ taxa usually exists in anaerobic and sulfur-rich extreme habitats (Campbell and Cary, 2004). In this study, the main taxa of the rTCA cycle pathway were Actinobacteria (mainly include Rubrobacteraceae and Pseudonocardiaceae, see Supplementary Figure S9B), Proteobacteria, Acidobacteria, and Cyanobacteria (Supplementary Figure S9A), which was consistent with previous studies (Hall et al., 2008). In the maize rhizosphere microbial community, *Rhodopseudomonas* and *Stappia* may be the main groups for carbon dioxide fixation through the rAcCoA pathway (Li et al., 2014). In this study, the main taxa in the rAcCoA pathway were Rubrobacteraceae and Pseudonocardiaceae (Supplementary Figure S10). In addition, *Rubrobacter radiotolerans*, which belong to Rubrobacteraceae was γ-irradiation and moderate thermophiles (Suzuki, 2015). Pseudonocardiaceae were found to be the main dominant taxa in the biological degradation of stony artifacts (Stomeo et al., 2008, 2009). Further, 3HP and 3HP/4HB decreased significantly with the increase in weathering time. The main group of 3HP has been reported as *Chloroflexus aurantiacus* (Holo, 1989). Previous studies have shown that 3HP is the main carbon fixation method in oolites (Diaz et al., 2014). The main taxa in the 3HP/4HB pathway are Crenarchaeota and Thaumarchaeota (Bergauer et al., 2013). To sum up, the types of microorganisms involved in the C fixation in carbonatite rock rind are similar to those in the previous studies, which are in line with their respective ecological amplitudes.

N is an essential component of all living things, and its availability depends on the various nitrogen conversion reactions carried out by microorganisms (Kuypers et al., 2018). For N cycling, nitrification, and denitrification decreased significantly with increasing weathering time, which may be related to nutrient availability (Sun and Badgley, 2019). The S-cycle related functional genes did not change significantly with the increase of weathering time, and the main taxa was Sphingomonadaceae and Rubrobacteraceae (Supplementary Figure S6).

Sphingomonadaceae and Rubrobacteraceae were more common on the surfaces of gravestones in all weathering time periods and were closely related to C, N and S cycle processes. Sphingomonadaceae are widespread in almost all types of natural environments and are usually Gram-negative bacteria, mostly containing carotenoids, which are yellow in their natural environment (Glaeser and Kämpfer, 2014). Some genera of Sphingomonadaceae (e.g., *Blastomonas*, *Sandaracinobacter*, etc.) are facultative prototrophs (Glaeser and Kämpfer, 2014). Bacteria able to produce growth factors are called prototrophs (D’Souza et al., 2018; Soto-Martin et al., 2020). These properties suggest that Sphingomonadaceae may be autotrophic and able to survive in extreme environments (e.g., rocky surfaces, polluted water bodies, etc.). In addition, some genera of the Sphingomonadaceae (e.g., *Sphingomonas*) have been found to have nitrogen-fixing functions. Besides, Several species of the genus *Rubrobacter* (Rubrobacteraceae) can tolerate extremely high levels of ionizing radiation and are moderately thermophilic or thermophilic (Albuquerque and da Costa, 2014). These species were primarily isolated mainly from deteriorated stone artefacts. In addition, as well as isolated from arid soils and dry, discolored, and sun-exposed walls (Albuquerque and da Costa, 2014). The species characteristics of Rubrobacteraceae described above all suggest that Rubrobacteraceae are suited to survive in arid, nutrient-poor environments and it is not surprising that they have been found to colonise the surface of tombstones in large numbers.

## Conclusion

This study provides new insights into how the ecological succession strategies and functional gene changes of bacterial, archaeal, and fungal communities vary with the time of carbonatite weathering. We have three main conclusions. First, during the weathering of carbonatite over time, microbial taxa gradually shifted from r-strategists to K-strategists, and the abundance of functional genes related to C and N was gradually increased. Secondly, the properties of carbonatite parent rock determine the colonization and distribution of microorganisms. In the early stage of carbonatite weathering, the weathering potential is large, the weathering rate is fast, and the weathering degree is small. Therefore, in the early stage, there are many kinds of microorganisms, but the abundance of genes related to element cycling is small, and the rate of environmental modification is fast, while the opposite is true in the later stage of weathering. Thirdly, the increase in the abundance of genes related to the N cycle promotes an increase in carbonatite weathering degree and drives the succession of carbonatite rock rind microorganisms. In summary, this study provides a coupling mechanism between carbonatite weathering rind microorganisms and potential functional genes during succession, and at the same time, we also reveal the coupling mechanism between carbonatite weathering degrees and potential functional genes.

## Data availability statement

The datasets presented in this study can be found in online repositories. The 36 Sequence data associated with this project have been deposited in the NCBI Short Read Archive database (Accession Number: PRJNA848062). The names of the repository/repositories and accession number(s) can be found in the article/Supplementary material.

## Author contributions

LYu conceived the project. JC, FL, XZ, YW, LZ, and LYan collected samples in the field. JC, FL, XZ and LYan performed data analysis; JC, FL, XZ, and YW performed the experiment. JC and LYu wrote the manuscript. All authors contributed to the article and approved the submitted version.

## Funding

This work was supported by the Project of National Key Research and Development Program of China (2016YFC0502604); Construction Program of Biology First-class Discipline in Guizhou (GNYL[2017]009); the Postgraduate Education Innovation Program in Guizhou Province (grant number YJSKYJJ[2021]079); and the Natural Science Research Project of Guizhou Provincial Department of Education (Qian Jiao He KY Zi [2018]170).

## Conflict of interest

The authors declare that the research was conducted in the absence of any commercial or financial relationships that could be construed as a potential conflict of interest.

## Publisher’s note

All claims expressed in this article are solely those of the authors and do not necessarily represent those of their affiliated organizations, or those of the publisher, the editors and the reviewers. Any product that may be evaluated in this article, or claim that may be made by its manufacturer, is not guaranteed or endorsed by the publisher.

## Footnotes

^1^ www.illumina.com

^2^ https://github.com/OpenGene/fastp

^3^ https://github.com/voutcn/megahit

^4^ http://metagene.cb.k.u-tokyo.ac.jp

^5^ http://www.ncbi.nlm.nih.gov/Taxonomy/taxonomyhome.html/index.cgi?chapter=tgencodes#SG1

^6^ http://www.bioinformatics.org/cd-hit/

^7^ http://soap.genomics.org.cn/

^8^ http://www.diamondsearch.org/index.php

^9^ http://www.diamondsearch.org/index.php

^10^ http://www.diamondsearch.org/index.php

^11^ http://www.genome.jp/keeg/

^12^ http://www.cma.gov.cn/
