# Supplementary material for: Change in composition and potential functional genes of microbial communities on carbonatite rinds with different weathering times
Source: Front Microbiol. 2022 Nov 1;13:1024672. doi: 10.3389/fmicb.2022.1024672 (PMC9663929; doi:10.3389/fmicb.2022.1024672)
Supplement: Supplementary file 1 [file Data_Sheet_1.pdf]

Table S1

Basic information about the samples (SN is sampling number, ST is the start time of the limestone weathering, WT is weathering time, SA is sampling area, SW is sampling weight, SWPA is sampling weight of per unit area, "\" indicates that data is missing).

SN	ST	WT (a)	Length (m)	Width (m)	Thickness (m)	SA (m^2)	SW (g)	SWPA ($g \cdot m^{-2}$)
S919	2002	19	1.22	0.8	0.14	1.28	25.85	20.13
S912	2001	20	1.39	0.81	0.15	0.98	21.78	22.3
S937	1999	22	1.2	0.74	0.19	0.59	27.38	46.13
S914	1998	23	1.22	0.72	0.15	0.84	31.5	37.61
S962	1998	23	1.39	0.78	0.19	0.86	51.73	60.47
S936	1987	34	1.34	0.77	0.17	0.56	15.29	27.55
S935	1984	37	1.3	0.74	0.19	0.8	29.55	36.81
S950	1984	37	1.14	0.7	0.16	0.72	21.38	29.52
S910	1982	39	1.2	0.74	0.19	0.59	18.38	31.1
S777	1974	47	1.07	0.67	0.16	0.47	13.06	28.07
S938	1963	58	1.12	0.64	0.13	0.47	15.21	32.06
S907	1949	72	1.41	0.69	0.14	0.61	20.27	33.17
S955	1945	76	1.2	0.6	0.16	0.56	63.48	113.11
S666	1933	88	1.16	0.63	0.16	0.82	17.88	21.73
S917	1917	104	1.35	0.72	0.17	0.64	19.62	30.63
S333	1888	133	1.37	0.73	0.13	0.9	11.4	12.65
S999	1860	161	1.28	0.67	0.19	0.6	16.2	27.07
S928	1808	213	0.99	0.51	0.09	\	113.92	\

Table S2 Details of the functional pathways (genes) associated with the C, N and S cycles and their total abundance.

Reference pathway 1	Reference pathway 2	KO	KEGG Name	Total abundance	KO Description
C Fixation	Hydroxypropionate-hydroxybutylate cycle (3HP/4HB)	K00626	E2.3.1.9, atoB	187290	acetyl-CoA C-acetyltransferase
C Fixation	Hydroxypropionate-hydroxybutylate cycle (3HP/4HB)	K01847	MUT	55472	methylmalonyl-CoA mutase
C Fixation	Hydroxypropionate-hydroxybutylate cycle (3HP/4HB)	K01961	accC	74386	acetyl-CoA carboxylase, biotin carboxylase subunit
C Fixation	Hydroxypropionate-hydroxybutylate cycle (3HP/4HB)	K01962	accA	46112	acetyl-CoA carboxylase carboxyl transferase subunit alpha
C Fixation	Hydroxypropionate-hydroxybutylate cycle (3HP/4HB)	K01963	accD	40980	acetyl-CoA carboxylase carboxyl transferase subunit beta
C Fixation	Hydroxypropionate-hydroxybutylate cycle (3HP/4HB)	K02160	accB, bccP	28712	acetyl-CoA carboxylase biotin carboxyl carrier protein
C Fixation	Hydroxypropionate-hydroxybutylate cycle (3HP/4HB)	K05606	MCEE, epi	34974	methylmalonyl-CoA/ethylmalonyl-CoA epimerase
C Fixation	Hydroxypropionate-hydroxybutylate cycle (3HP/4HB)	K14465	K14465	310	succinate semialdehyde reductase (NADPH)
C Fixation	Hydroxypropionate-hydroxybutylate cycle (3HP/4HB)	K14469	K14469	418	acrylyl-CoA reductase (NADPH) / 3-hydroxypropionyl-CoA dehydratase / 3-hydroxypropionyl-CoA synthetase
C Fixation	Hydroxypropionate-hydroxybutylate cycle (3HP/4HB)	K14534	abfD	3390	4-hydroxybutyryl-CoA dehydratase / vinylacetyl-CoA-Delta-isomerase
C Fixation	Hydroxypropionate-hydroxybutylate cycle (3HP/4HB)	K15016	K15016	4686	enoyl-CoA hydratase / 3-hydroxyacyl-CoA dehydrogenase
C Fixation	Hydroxypropionate-hydroxybutylate cycle (3HP/4HB)	K15019	K15019	1326	3-hydroxypropionyl-coenzyme A dehydratase
C Fixation	Hydroxypropionate-hydroxybutylate cycle (3HP/4HB)	K15020	K15020	14	acryloyl-coenzyme A reductase
C Fixation	Hydroxypropionate-hydroxybutylate cycle (3HP/4HB)	K15052	K15052	1676	propionyl-CoA carboxylase
C Fixation	Hydroxypropionate-hydroxybutylate cycle (3HP/4HB)	K18594	K18594	448	3-hydroxypropionyl-CoA synthetase (ADP-forming)
C Fixation	Hydroxypropionate-hydroxybutylate cycle (3HP/4HB)	K18602	K18602	76	malonic semialdehyde reductase
C Fixation	Hydroxypropionate-hydroxybutylate cycle (3HP/4HB)	K18603	K18603	134	acetyl-CoA/propionyl-CoA carboxylase
C Fixation	Hydroxypropionate-hydroxybutylate cycle (3HP/4HB)	K18604	K18604	92	acetyl-CoA/propionyl-CoA carboxylase
C Fixation	Hydroxypropionate-hydroxybutylate cycle (3HP/4HB)	K18605	K18605	82	biotin carboxyl carrier protein
C Fixation	3-Hydroxypropionate bi-cycle (3HP)	K00239	sdhA, frdA	101606	succinate dehydrogenase / fumarate reductase, flavoprotein subunit
C Fixation	3-Hydroxypropionate bi-cycle (3HP)	K00244	frdA	11146	fumarate reductase flavoprotein subunit
C Fixation	3-Hydroxypropionate bi-cycle (3HP)	K01676	E4.2.1.2A, fumA, fumB	16072	fumarate hydratase, class I
C Fixation	3-Hydroxypropionate bi-cycle (3HP)	K01677	E4.2.1.2AA, fumA	6420	fumarate hydratase subunit alpha
C Fixation	3-Hydroxypropionate bi-cycle (3HP)	K01678	E4.2.1.2AB, fumB	4722	fumarate hydratase subunit beta
C Fixation	3-Hydroxypropionate bi-cycle (3HP)	K01903	sucC	70918	succinyl-CoA synthetase beta subunit
C Fixation	3-Hydroxypropionate bi-cycle (3HP)	K09709	meh	10834	3-methylfumaryl-CoA hydratase
C Fixation	3-Hydroxypropionate bi-cycle (3HP)	K14449	mch, mcd	6092	2-methylfumaryl-CoA hydratase
C Fixation	3-Hydroxypropionate bi-cycle (3HP)	K14470	mct	5838	2-methylfumaryl-CoA isomerase
C Fixation	3-Hydroxypropionate bi-cycle (3HP)	K14471	smtA1	44	succinyl-CoA:(S)-malate CoA-transferase subunit A
C Fixation	3-Hydroxypropionate bi-cycle (3HP)	K14472	smtB	74	succinyl-CoA:(S)-malate CoA-transferase subunit B
C Fixation	3-Hydroxypropionate bi-cycle (3HP)	K18209	tfrA	76	fumarate reductase (CoM/CoB) subunit A
C Fixation	Dicarboxylate - hydroxybutyrate cycle (DC/4HB)	K00024	mdh	57346	malate dehydrogenase
C Fixation	Dicarboxylate - hydroxybutyrate cycle (DC/4HB)	K00169	porA	1140	pyruvate ferredoxin oxidoreductase alpha subunit
C Fixation	Dicarboxylate - hydroxybutyrate cycle (DC/4HB)	K00170	porB	1280	pyruvate ferredoxin oxidoreductase beta subunit
C Fixation	Dicarboxylate - hydroxybutyrate cycle (DC/4HB)	K00171	porD	1410	pyruvate ferredoxin oxidoreductase delta subunit
C Fixation	Dicarboxylate - hydroxybutyrate cycle (DC/4HB)	K00172	porC, porG	64	pyruvate ferredoxin oxidoreductase gamma subunit
C Fixation	Dicarboxylate - hydroxybutyrate cycle (DC/4HB)	K01006	ppdK	73022	pyruvate, orthophosphate dikinase
C Fixation	Dicarboxylate - hydroxybutyrate cycle (DC/4HB)	K01007	pps, ppsA	57910	pyruvate, water dikinase
C Fixation	Dicarboxylate - hydroxybutyrate cycle (DC/4HB)	K01595	ppc	82742	phosphoenolpyruvate carboxylase
C Fixation	Dicarboxylate - hydroxybutyrate cycle (DC/4HB)	K01958	PC, pyc	41486	pyruvate carboxylase
C Fixation	Dicarboxylate - hydroxybutyrate cycle (DC/4HB)	K01959	pycA	144	pyruvate carboxylase subunit A
C Fixation	Dicarboxylate - hydroxybutyrate cycle (DC/4HB)	K01960	pycB	70	pyruvate carboxylase subunit B
C Fixation	Dicarboxylate - hydroxybutyrate cycle (DC/4HB)	K03737	por, nifJ	2234	pyruvate-ferredoxin/flavodoxin oxidoreductase
C Fixation	Reductive citrate cycle (rTCA)	K00031	IDH1, IDH2, icd	64694	isocitrate dehydrogenase
C Fixation	Reductive citrate cycle (rTCA)	K00177	korC, oorC	86	2-oxoglutarate ferredoxin oxidoreductase subunit gamma
C Fixation	Reductive citrate cycle (rTCA)	K01648	ACLY	842	ATP citrate (pro-S)-lyase
C Fixation	Reductive citrate cycle (rTCA)	K01681	ACO, acnA	126610	aconitate hydratase
C Fixation	Reductive citrate cycle (rTCA)	K01682	acnB	2482	aconitate hydratase 2 / 2-methylisocitrate dehydratase
C Fixation	Reductive citrate cycle (rTCA)	K15231	aclB	18	ATP-citrate lyase beta-subunit
C Fixation	Reductive citrate cycle (rTCA)	K15232	ccsA	1576	citryl-CoA synthetase large subunit
C Fixation	Reductive citrate cycle (rTCA)	K15234	ccl	256	citryl-CoA lyase
C Fixation	Reductive acetyl-CoA or Wood-Ljungdahl pathway (rAcCoA)	K00148	fdhA	47214	glutathione-independent formaldehyde dehydrogenase
C Fixation	Reductive acetyl-CoA or Wood-Ljungdahl pathway (rAcCoA)	K00297	metF, MTHFR	83184	methylenetetrahydrofolate reductase (NADPH)
C Fixation	Reductive acetyl-CoA or Wood-Ljungdahl pathway (rAcCoA)	K01491	folD	60076	methylenetetrahydrofolate dehydrogenase (NADP+) / methenyltetrahydrofolate cyclohydrolase
C Fixation	Reductive acetyl-CoA or Wood-Ljungdahl pathway (rAcCoA)	K01938	fhs	29612	formate--tetrahydrofolate ligase
C Fixation	Calvin cycle	K00134	GAPDH, gapA	63076	glyceraldehyde 3-phosphate dehydrogenase
C Fixation	Calvin cycle	K00150	gap2	4520	glyceraldehyde-3-phosphate dehydrogenase (NAD(P))
C Fixation	Calvin cycle	K00615	E2.2.1.1, tktA, tktB	172896	transketolase
C Fixation	Calvin cycle	K00855	PRK, prkB	19732	phosphoribulokinase
C Fixation	Calvin cycle	K00927	PGK, pgk	67880	phosphoglycerate kinase
C Fixation	Calvin cycle	K01086	fbp-SEBP	364	fructose-1,6-bisphosphatase I / sedoheptulose-1,7-bisphosphatase
C Fixation	Calvin cycle	K01100	E3.1.3.37	118	sedoheptulose-bisphosphatase

Reference pathway 1	Reference pathway 2	KO	KEGG Name	Total abundance	KO Description
C Fixation	Calvin cycle	K01601	rbcL	23686	ribulose-bisphosphate carboxylase large chain
C Fixation	Calvin cycle	K01602	rbcS	6470	ribulose-bisphosphate carboxylase small chain
C Fixation	Calvin cycle	K01622	K01622	402	fructose 1,6-bisphosphate aldolase/phosphatase
C Fixation	Calvin cycle	K01623	ALDO	21546	fructose-bisphosphate aldolase, class I
C Fixation	Calvin cycle	K01624	FBA, fbaA	46766	fructose-bisphosphate aldolase, class II
C Fixation	Calvin cycle	K01807	rpiA	25928	ribose 5-phosphate isomerase A
C Fixation	Calvin cycle	K01808	rpiB	38038	ribose 5-phosphate isomerase B
C Fixation	Calvin cycle	K02446	glpX	34020	fructose-1,6-bisphosphatase II
C Fixation	Calvin cycle	K03841	FBP, fbp	17612	fructose-1,6-bisphosphatase I
C Fixation	Calvin cycle	K05298	GAPA	1156	glyceraldehyde-3-phosphate dehydrogenase (NADP+) (phosphorylating)
C Fixation	Calvin cycle	K11532	glpX-SEBP	15456	fructose-1,6-bisphosphatase II / sedoheptulose-1,7-bisphosphatase
C Degradation	Hemicellulose	K01181	E3.2.1.8, xynA	22506	endo-1,4-beta-xylanase
C Degradation	Hemicellulose	K01209	abfA	47658	alpha-L-arabinofuranosidase
C Degradation	Hemicellulose	K06113	abnA	20904	arabinan endo-1,5-alpha-L-arabinosidase
C Degradation	Hemicellulose	K15531	rexA	462	oligosaccharide reducing-end xylanase
C Degradation	Hemicellulose	K15924	xynC	216	glucuronoarabinoxylan endo-1,4-beta-xylanase
C Degradation	Hemicellulose	K18205	hypBA1	6	non-reducing end beta-L-arabinofuranosidase
C Degradation	Hemicellulose	K20844	abf1	776	non-reducing end alpha-L-arabinofuranosidase
C Degradation	Starch	K01176	AMY, amyA, malS	24570	alpha-amylase
C Degradation	Starch	K01178	SGA1	14748	glucoamylase
C Degradation	Starch	K01200	pulA	770	pullulanase
C Degradation	Starch	K01208	cd, ma, nplT	13574	cyclomaltodextrinase / maltogenic alpha-amylase / neopullulanase
C Degradation	Starch	K05343	treS	72384	maltose alpha-D-glucosyltransferase / alpha-amylase
C Degradation	Starch	K07405	E3.2.1.1A	676	alpha-amylase
C Degradation	Starch	K12047	MGAM	16	maltase-glucoamylase
C Degradation	Starch	K21575	susA	10424	neopullulanase
C Degradation	Aromatics	K10533	E3.3.2.8	2444	limonene-1,2-epoxide hydrolase
C Degradation	Aromatics	K21802	vdh	808	vanillin dehydrogenase
C Degradation	Chintin	K01183	E3.2.1.14	14994	chitinase
C Degradation	Chintin	K01205	NAGLU	466	alpha-N-acetylglucosaminidase
C Degradation	Chintin	K01227	ENGASE	390	mannosyl-glycoprotein endo-beta-N-acetylglucosaminidase
C Degradation	Chintin	K03791	K03791	10246	putative chitinase
C Degradation	Chintin	K17525	CHID1	20	chitinase domain-containing protein 1
C Degradation	Chintin	K20547	CHIB	470	basic endochitinase B
C Degradation	Chintin	K23989	lytD, lytB	1000	mannosyl-glycoprotein endo-beta-N-acetylglucosaminidase
C Degradation	Lignin	K00422	E1.10.3.1	34	polyphenol oxidase
C Degradation	Lignin	K20205	mpn	66	manganese peroxidase
C Degradation	Cellulose	K01179	E3.2.1.4	69354	endoglucanase
C Degradation	Cellulose	K20542	bcsZ	4608	endoglucanase
Methane Metabolism	Methane Oxidation	K10944	pmoA-amoA	398	methane/ammonia monooxygenase subunit A
Methane Metabolism	Methane Oxidation	K10945	pmoB-amoB	512	methane/ammonia monooxygenase subunit B
Methane Metabolism	Methane Oxidation	K10946	pmoC-amoC	592	methane/ammonia monooxygenase subunit C
Methane Metabolism	Methane Oxidation	K14028	mdh1, mxaF	18	methanol dehydrogenase (cytochrome c) subunit 1
Methane Metabolism	Methane Oxidation	K16157	mmoX	22	methane monooxygenase component A alpha chain
Methane Metabolism	Methane Oxidation	K23995	xoxF	1316	lanthanide-dependent methanol dehydrogenase
Methane Metabolism	Methanogenesis	K00200	fwdA, fmdA	6518	formylmethanofuran dehydrogenase subunit A
Methane Metabolism	Methanogenesis	K00201	fwdB, fmdB	5512	formylmethanofuran dehydrogenase subunit B
Methane Metabolism	Methanogenesis	K00202	fwdC, fmdC	4932	formylmethanofuran dehydrogenase subunit C
Methane Metabolism	Methanogenesis	K00203	fwdD, fmdD	52	formylmethanofuran dehydrogenase subunit D
Methane Metabolism	Methanogenesis	K00320	mer	28628	5,10-methylenetetrahydromethanopterin reductase
Methane Metabolism	Methanogenesis	K00577	mtrA	250	tetrahydromethanopterin S-methyltransferase subunit A
Methane Metabolism	Methanogenesis	K00625	E2.3.1.8, pta	8922	phosphate acetyltransferase
Methane Metabolism	Methanogenesis	K00672	ftr	6898	formylmethanofuran--tetrahydromethanopterin N-formyltransferase
Methane Metabolism	Methanogenesis	K00925	ackA	37202	acetate kinase
Methane Metabolism	Methanogenesis	K01499	mch	6258	methenyltetrahydromethanopterin cyclohydrolase
Methane Metabolism	Methanogenesis	K01895	ACSS1_2, acs	158670	acetyl-CoA synthetase
Methane Metabolism	Methanogenesis	K08264	hdrD	8098	heterodisulfide reductase subunit D
Methane Metabolism	Methanogenesis	K11261	fwdE, fmdE	2160	formylmethanofuran dehydrogenase subunit E
Methane Metabolism	Methanogenesis	K14083	mttB	18632	trimethylamine---corrinoid protein Co-methyltransferase
Methane Metabolism	Methanogenesis	K14127	mvhD, vhuD, vhcD	80	F420-non-reducing hydrogenase iron-sulfur subunit
Methane Metabolism	Methanogenesis	K22516	fdhA	262	formate dehydrogenase (coenzyme F420) alpha subunit
Assimilatory N Reduction (ANRA)	null	K00360	nasB	368	assimilatory nitrate reductase electron transfer subunit

Reference pathway 1	Reference pathway 2	KO	KEGG Name	Total abundance	KO Description
Assimilatory N Reduction (ANRA)	null	K00366	nirA	26702	ferredoxin-nitrite reductase
Assimilatory N Reduction (ANRA)	null	K00367	narB	11682	ferredoxin-nitrate reductase
Assimilatory N Reduction (ANRA)	null	K00372	nasA	27656	assimilatory nitrate reductase catalytic subunit
Assimilatory N Reduction (ANRA)	null	K10534	NR	1196	nitrate reductase (NAD(P)H)
Assimilatory N Reduction (ANRA)	null	K15879	narC	1714	cytochrome b-561
Assimilatory N Reduction (ANRA)	null	K17877	NIT-6	1080	nitrite reductase (NAD(P)H)
Dissimilatory N Reduction (DNRA)	null	K00362	nirB	36226	nitrite reductase (NADH) large subunit
Dissimilatory N Reduction (DNRA)	null	K00363	nirD	9200	nitrite reductase (NADH) small subunit
Dissimilatory N Reduction (DNRA)	null	K03385	nrfA	874	nitrite reductase (cytochrome c-552)
Dissimilatory N Reduction (DNRA)	null	K15876	nrfH	362	cytochrome c nitrite reductase small subunit
N Fixation	null	K02586	nifD	334	nitrogenase molybdenum-iron protein alpha chain
N Fixation	null	K02588	nifH	770	nitrogenase iron protein NifH
N Fixation	null	K02591	nifK	378	nitrogenase molybdenum-iron protein beta chain
N Fixation	null	K02595	nifW	92	nitrogenase-stabilizing/protective protein
N Fixation	null	K22896	vnfD	442	vanadium-dependent nitrogenase alpha chain
N Fixation	null	K22897	vnfK	178	vanadium-dependent nitrogenase beta chain
N Transport	null	K15576	nrtA, nasF, cynA	20044	nitrate/nitrite transport system substrate-binding protein
N Transport	null	K15577	nrtB, nasE, cynB	15350	nitrate/nitrite transport system permease protein
N Transport	null	K15578	nrtC, nasD	18190	nitrate/nitrite transport system ATP-binding protein
N Transport	null	K15579	nrtD, cynD	250	nitrate/nitrite transport system ATP-binding protein
Denitrification	null	K00368	nirK	1942	nitrite reductase (NO-forming)
Denitrification	null	K00370	narG, narZ, nxrA	3436	nitrate reductase / nitrite oxidoreductase, alpha subunit
Denitrification	null	K00371	narH, narY, nxrB	1616	nitrate reductase / nitrite oxidoreductase, beta subunit
Denitrification	null	K00373	narJ, narW	1048	nitrate reductase molybdenum cofactor assembly chaperone NarJ/NarW
Denitrification	null	K00374	narI, narV	792	nitrate reductase gamma subunit
Denitrification	null	K00376	nosZ	718	nitrous-oxide reductase
Denitrification	null	K02305	norC	780	nitric oxide reductase subunit C
Denitrification	null	K02567	napA	320	nitrate reductase (cytochrome)
Denitrification	null	K02568	napB	12	nitrate reductase (cytochrome), electron transfer subunit
Denitrification	null	K02569	napC	130	cytochrome c-type protein NapC
Denitrification	null	K04561	norB	3232	nitric oxide reductase subunit B
Denitrification	null	K15864	nirS	440	nitrite reductase (NO-forming) / hydroxylamine reductase
Nitrification	null	K10535	hao	74	hydroxylamine dehydrogenase
Nitrification	null	K10944	pmoA-amoA	398	methane/ammonia monooxygenase subunit A
Nitrification	null	K10945	pmoB-amoB	512	methane/ammonia monooxygenase subunit B
Nitrification	null	K10946	pmoC-amoC	592	methane/ammonia monooxygenase subunit C
Organic N Metabolism (ONM)	null	K00260	gudB, rocG	126	glutamate dehydrogenase
Organic N Metabolism (ONM)	null	K00261	GLUD1_2, gdhA	33602	glutamate dehydrogenase (NAD(P)+)
Organic N Metabolism (ONM)	null	K00262	E1.4.1.4, gdhA	7198	glutamate dehydrogenase (NADP+)
Organic N Metabolism (ONM)	null	K00264	GLT1	1170	glutamate synthase (NADH)
Organic N Metabolism (ONM)	null	K00265	gltB	133450	glutamate synthase (NADPH) large chain
Organic N Metabolism (ONM)	null	K00266	gltD	51934	glutamate synthase (NADPH) small chain
Organic N Metabolism (ONM)	null	K01424	E3.5.1.1, ansA, ansB	28372	L-asparaginase
Organic N Metabolism (ONM)	null	K01425	glsA, GLS	29986	glutaminase
Organic N Metabolism (ONM)	null	K01428	ureC	46298	urease subunit alpha
Organic N Metabolism (ONM)	null	K01429	ureB	10374	urease subunit beta
Organic N Metabolism (ONM)	null	K01430	ureA	7148	urease subunit gamma
Organic N Metabolism (ONM)	null	K01915	glnA, GLUL	213852	glutamine synthetase
Organic N Metabolism (ONM)	null	K01953	asnB, ASNS	205160	asparagine synthase (glutamine-hydrolysing)
Organic N Metabolism (ONM)	null	K05597	aspQ, ansB, ansA	138	glutamin-(asparagin-)ase
Organic N Metabolism (ONM)	null	K15371	GDH2	104696	glutamate dehydrogenase
Assimilatory sulfate reduction (ASR)	null	K00380	cysJ	16086	sulfite reductase (NADPH) flavoprotein alpha-component
Assimilatory sulfate reduction (ASR)	null	K00381	cysI	19832	sulfite reductase (NADPH) hemoprotein beta-component
Assimilatory sulfate reduction (ASR)	null	K00390	cysH	45230	phosphoadenosine phosphosulfate reductase
Assimilatory sulfate reduction (ASR)	null	K00392	sir	41706	sulfite reductase (ferredoxin)
Assimilatory sulfate reduction (ASR)	null	K00640	cysE	35630	serine O-acetyltransferase
Assimilatory sulfate reduction (ASR)	null	K00860	cysC	25346	adenylylsulfate kinase
Assimilatory sulfate reduction (ASR)	null	K00955	cysNC	36830	bifunctional enzyme CysN/CysC
Assimilatory sulfate reduction (ASR)	null	K00956	cysN	18100	sulfate adenylyltransferase subunit 1
Assimilatory sulfate reduction (ASR)	null	K00957	cysD	37940	sulfate adenylyltransferase subunit 2
Assimilatory sulfate reduction (ASR)	null	K00958	sat, met3	32660	sulfate adenylyltransferase
Assimilatory sulfate reduction (ASR)	null	K01082	cysQ, MET22, BPNT1	35138	3'(2'), 5'-bisphosphate nucleotidase

Reference pathway 1	Reference pathway 2	KO	KEGG Name	Total abundance	KO Description
Assimilatory sulfate reduction (ASR)	null	K01738	cysK	68768	cysteine synthase
Assimilatory sulfate reduction (ASR)	null	K02045	cysA	36928	sulfate/thiosulfate transport system ATP-binding protein
Assimilatory sulfate reduction (ASR)	null	K02046	cysU	32842	sulfate/thiosulfate transport system permease protein
Assimilatory sulfate reduction (ASR)	null	K02047	cysW	29574	sulfate/thiosulfate transport system permease protein
Assimilatory sulfate reduction (ASR)	null	K02048	cysP	2938	sulfate/thiosulfate transport system substrate-binding protein
Assimilatory sulfate reduction (ASR)	null	K13811	PAPSS	130	3'-phosphoadenosine 5'-phosphosulfate synthase
Dissimilatory sulfate reduction (DSR)	null	K00394	aprA	196	adenylylsulfate reductase, subunit A
Dissimilatory sulfate reduction (DSR)	null	K00395	aprB	28	adenylylsulfate reductase, subunit B
Dissimilatory sulfate reduction (DSR)	null	K04085	tusA, sirA	3458	tRNA 2-thiouridine synthesizing protein A
Dissimilatory sulfate reduction (DSR)	null	K11179	tusE, dsrC	2	tRNA 2-thiouridine synthesizing protein E
Thiosulfate oxidation by SOX complex (SOX)	null	K17222	soxA	6068	L-cysteine S-thiosulfotransferase
Thiosulfate oxidation by SOX complex (SOX)	null	K17223	soxX	3066	L-cysteine S-thiosulfotransferase
Thiosulfate oxidation by SOX complex (SOX)	null	K17224	soxB	1252	S-sulfosulfanyl-L-cysteine sulfohydrolase
Thiosulfate oxidation by SOX complex (SOX)	null	K17225	soxC	2992	sulfane dehydrogenase subunit SoxC
Thiosulfate oxidation by SOX complex (SOX)	null	K17226	soxY	12314	sulfur-oxidizing protein SoxY
Thiosulfate oxidation by SOX complex (SOX)	null	K17227	soxZ	2632	sulfur-oxidizing protein SoxZ
Thiosulfate oxidation by SOX complex (SOX)	null	K22622	soxD	2502	S-disulfanyl-L-cysteine oxidoreductase SoxD

Table S3

The variations of the Level3 of KEGG over different weathering times (relative abundance>1%). The relative abundances of functional genes were averaged for each site (n = 18). The variance explained (R^2), regression slope and p value of the linear regression with different weathering times were shown in the table (* indicates $p < 0.05$, ** indicates $p < 0.01$ and ***indicates $p < 0.001$).

Level 3	Relative abundance % (mean abundance)	Slope	SE	R^2	p value
Biosynthesis of secondary metabolites	8.41% (1114841)	9.2E-05	3.8E-05	0.27	0.027*
Microbial metabolism in diverse environments	5.67% (750945)	1.3E-04	5.5E-05	0.27	0.027*
Biosynthesis of amino acids	3.05% (403951)	1.6E-04	5.5E-05	0.35	0.010**
Carbon metabolism	2.89% (382342)	1.7E-04	4.6E-05	0.47	0.002**
ABC transporters	2.73% (362244)	-4.2E-04	1.2E-04	0.43	0.003**
Quorum sensing	1.80% (239966)	-3.4E-04	1.1E-04	0.39	0.006**
Two-component system	1.59% (210842)	-2.6E-04	1.0E-04	0.30	0.019*
Purine metabolism	1.37% (181684)	-1.9E-06	5.8E-05	0.00	0.974
Glyoxylate and dicarboxylate metabolism	1.13% (149040)	2.9E-04	9.2E-05	0.39	0.006**
Oxidative phosphorylation	1.11% (146880)	1.2E-05	5.6E-05	0.00	0.840
Pyruvate metabolism	1.03% (136109)	2.6E-04	4.4E-05	0.68	<0.001***
Amino sugar and nucleotide sugar metabolism	1.02% (135690)	-4.5E-05	6.7E-05	0.03	0.511

Table S4

The variations of the Cazy annotation results over different weathering times (relative abundance>1%). The relative abundances of functional genes were averaged for each site (n = 18). The variance explained (R^2), regression slope and p value of the linear regression with different weathering times were shown in the table (AA: Auxiliary Activities; CBM: Carbohydrate-Binding Modules; CE: Carbohydrate Esterases; GH: Glycoside Hydrolases; GT: Glycosyl Transferases; PL: Polysaccharide Lyases).

Class	Relative abundance % (mean abundance)	Slope	SE	R^2	p value
CE	16.5% (118262)	1.80E-05	1.13E-04	0.002	0.875
CBM	2.11% (14938)	9.36E-05	2.13E-04	0.012	0.666
PL	2.47% (17497)	1.36E-05	1.81E-04	0.000	0.941
GH	30.93% (220039)	1.39E-05	8.17E-05	0.002	0.867
GT	38.39% (273639)	-6.63E-05	7.57E-05	0.046	0.394
AA	9.6% (67974)	2.07E-04	1.28E-04	0.140	0.127
