# Supplementary material for: Change in composition and potential functional genes of microbial communities on carbonatite rinds with different weathering times
Source: Front Microbiol. 2022 Nov 1;13:1024672. doi: 10.3389/fmicb.2022.1024672 (PMC9663929; doi:10.3389/fmicb.2022.1024672)
Supplement: Supplementary file 2 [file Image_1.PDF]

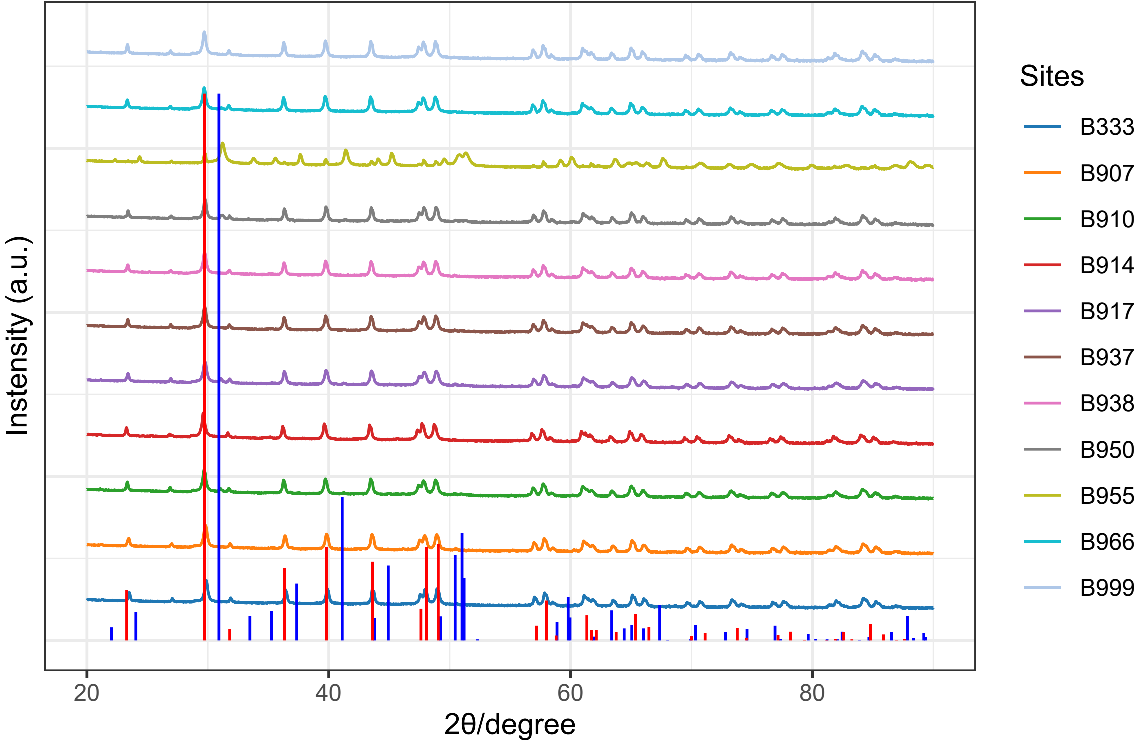

Fig S1. X-ray diffraction of carbonatite parent rock samples.
Note: The red vertical line indicates MgCaCO3 and the blue vertical line indicates CaMg(CO3)2
