# Supplementary material for: Change in composition and potential functional genes of microbial communities on carbonatite rinds with different weathering times
Source: Front Microbiol. 2022 Nov 1;13:1024672. doi: 10.3389/fmicb.2022.1024672 (PMC9663929; doi:10.3389/fmicb.2022.1024672)
Supplement: Supplementary file 3 [file Image_2.PDF]

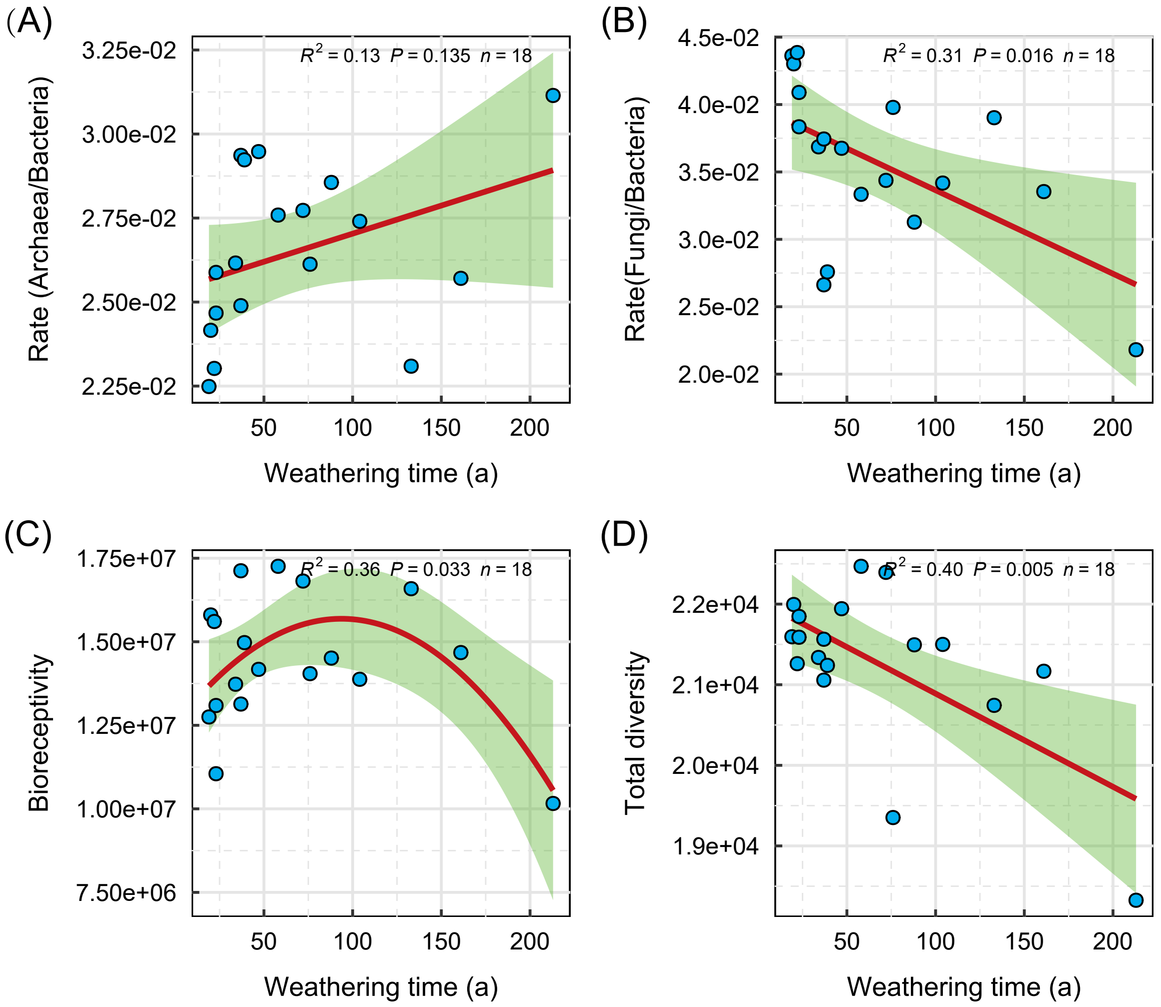

Fig S2. Regression fitting trend of microorganisms and weathering time. (A) The ratio of the relative abundance of archaea to bacteria. (B) The ratio of the relative abundance of fungi to bacteria. (C) Trends in bioreceptivity versus weathering time expressed as the number of all individuals in a single sample. (D) Regression fitting of microbial diversity (number of different kinds of species per sample) and weathering time in carbonatite regolith.
