# Supplementary material for: Change in composition and potential functional genes of microbial communities on carbonatite rinds with different weathering times
Source: Front Microbiol. 2022 Nov 1;13:1024672. doi: 10.3389/fmicb.2022.1024672 (PMC9663929; doi:10.3389/fmicb.2022.1024672)
Supplement: Supplementary file 12 [file Image_11.PDF]

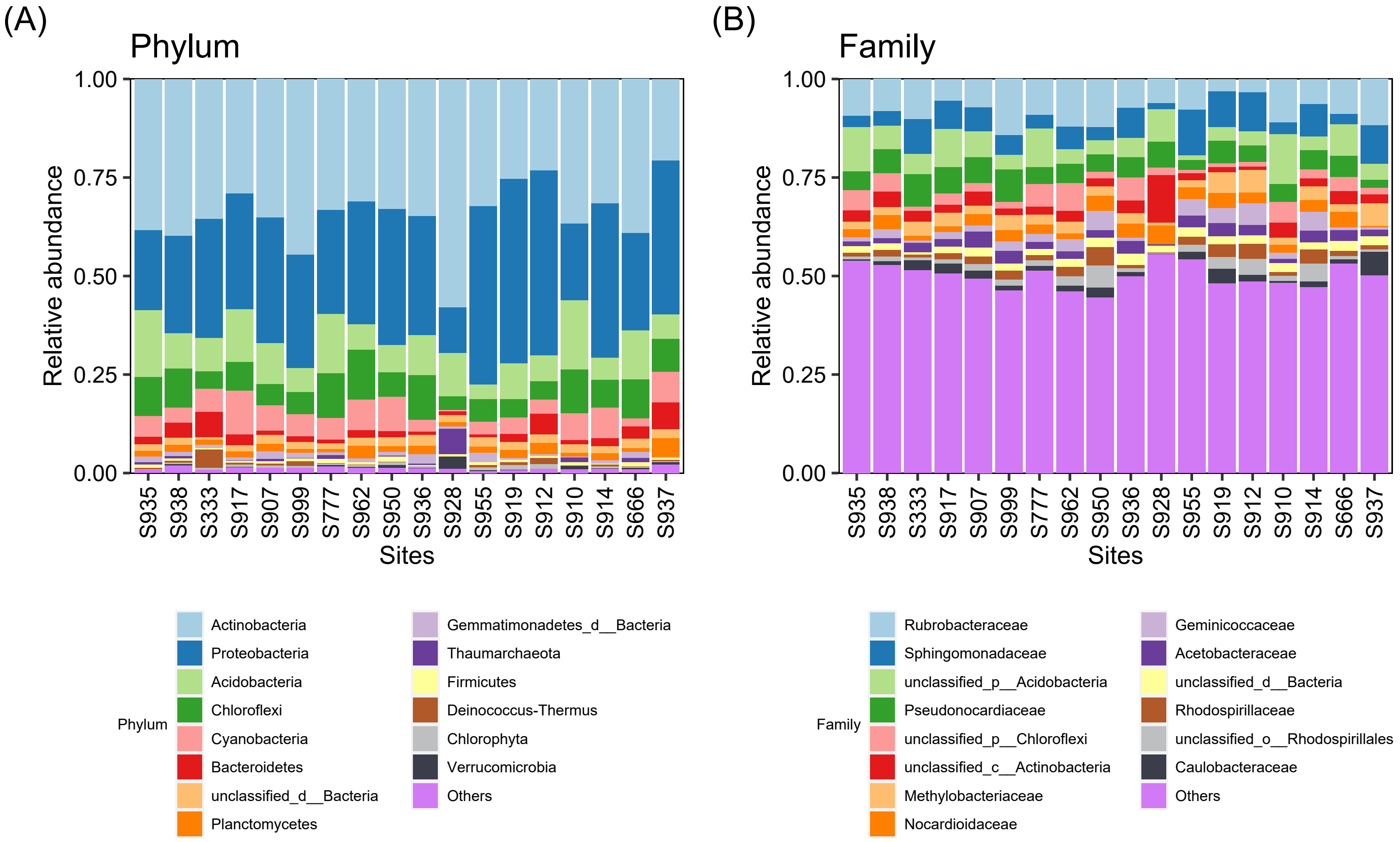

Fig S11. Relative abundance of taxa associated with the 3HP cycle pathway at the phylum (A) and family (B) taxonomic levels.
